# Scutellarin ameliorates ischemia/reperfusion-mediated endothelial dysfunction by upregulating cathepsin D expression to rescue autophagy-lysosomal function

**DOI:** 10.3389/fphar.2025.1538697

**Published:** 2025-03-03

**Authors:** Qizhen Zhuang, Lu Chen, Wanqian Wu, Qing Wang, Chunmin Kang, Yujuan Xiong, Xianzhang Huang

**Affiliations:** ^1^ The Second Clinical Medical College, Guangzhou University of Chinese Medicine, Guangzhou, China; ^2^ Department of Laboratory Medicine, The Second Affiliated Hospital of Guangzhou University of Chinese Medicine, Guangdong Provincial Key Laboratory of Research on Emergency in TCM, Guangzhou, China; ^3^ Department of Laboratory Medicine, Panyu Hospital of Chinese Medicine, Guangzhou University of Chinese Medicine, Guangzhou, China

**Keywords:** ischemia/reperfusion injury, endothelial dysfunction, scutellarin, autophagy-lysosomal function, cathepsin D

## Abstract

**Background:**

Endothelial dysfunction-induced microcirculation impairment and the no-reflow phenomenon are the leading causes of cardiac ischemia/reperfusion (I/R) injury. There is an urgent need to elucidate the underlying mechanism of I/R-mediated endothelial dysfunction and to identify effective drugs for treatment. Scutellarin (SCU), a flavonoid compound, has been extensively studied because of its various pharmacological properties, including its potent protective effects on the cardiovascular system. However, the anti-endothelial dysfunction efficacy and mechanisms of action of SCU have not been investigated.

**Approach and results:**

An *in vivo* I/R injury model was established using coronary artery ligation and release. An oxygen-glucose deprivation/oxygen-glucose resupply (OGD/OGR) approach was used to establish an *in vitro* I/R injury model. We evaluated the effects of SCU on endothelial dysfunction under I/R conditions, both *in vivo* and *in vitro*. SCU pretreatment promoted vasodilation and reperfusion of blood flow, inhibited myocardial injury and infarction, and improved cardiac function in I/R rats. Additionally, SCU inhibited cell membrane damage, reactive oxygen species (ROS) accumulation, inflammation, nitric oxide (NO) reduction, endothelin 1 (ET-1) elevation and increase in the expression levels of vascular endothelial growth factor (VEGF) and von willebrand factor (vWF) in endothelial cells. Mechanistically, SCU rescued the lysosomal flow and autophagic flux disrupted by I/R through upregulating cathepsin D (CTSD) levels. Knockdown of *CTSD* or treatment with the CTSD inhibitor pepstatin A (P.A) abrogated the protective effects of SCU on endothelial cells under I/R conditions.

**Conclusion:**

We demonstrated that SCU, via upregulation of CTSD levels in endothelial cells, rescued autophagy-lysosomal function and alleviated I/R-mediated endothelial dysfunction. Thus, SCU is a potential therapeutic drug for the prevention and treatment of cardiac I/R injury.

## 1 Introduction

Acute myocardial infarction (AMI) is an acute and critical cardiovascular disease with a high mortality rate. The myocardial infarction area is a decisive factor affecting the prognosis of patients with AMI. Timely percutaneous coronary intervention (PCI) to re-establish blood flow to the ischemic myocardium is the primary therapeutic approach for patients with AMI. However, despite the increasing utilization and success rates of PCI in clinical practice, a significant proportion of patients with AMI still experience pathological phenomena of cardiac structural damage and exacerbation of post-procedure functional impairments, which can lead to arrhythmia, sudden death, and other adverse cardiac events. This phenomenon is known as cardiac ischemia/reperfusion (I/R) injury. This is because, although the coronary arteries are recanalized in patients with AMI after reperfusion therapy, I/R-mediated endothelial dysfunction leads to impaired microcirculation and the no-reflow phenomenon, which ultimately causes additional damage to the myocardial tissue (Lawton et al., 2021).

Endothelial cells are situated on the inner surface of blood vessels and are responsible for maintaining the structural integrity of the blood vessel surface, controlling blood vessel tension, regulating angiogenesis, and maintaining microcirculation. During I/R, the massive production of reactive oxygen species (ROS) induces mitochondrial permeability transition pore (mPTP) opening and Ca^2+^ overload (Adameova et al., 2022; Rusciano et al., 2019). This causes the mitochondria to swell and impairs their function, which in turn triggers inflammasomes and activates inflammatory responses, resulting in endothelial dysfunction (Wang et al., 2020). Furthermore, the substantial generation of ROS in endothelial cells can lead to a reduction in nitric oxide (NO) levels and an elevation of endothelin-1 (ET-1) levels, which contribute to further endothelial dysfunction, resulting in a disruption of the normal regulatory mechanisms of vascular constriction and dilation. Timely removal of excess ROS is considered an effective strategy for inhibiting I/R-mediated endothelial dysfunction (Amruta and Bix, 2021). Numerous studies have shown that massive ROS production and damaged mitochondria during I/R can serve as signals to initiate autophagy, and activated autophagy can combat I/R injury by promptly eliminating the proteins and mitochondria damaged by ROS (Tong et al., 2020). However, when autophagy is blocked, cell damage is exacerbated (Zhuang et al., 2023).

Currently, there are no approved pharmacological therapies approved for I/R-mediated endothelial dysfunction. Studies have shown that intravenous infusion of tirofiban hydrochloride (TH) before PCI can improve coronary artery reperfusion and promote recovery of cardiac function (Sun et al., 2016). However, since TH can inhibit platelet aggregation, adverse reactions such as bleeding can easily occur when combined with TH treatment. Hence, there is an urgent need to identify safer and more efficient medications to treat I/R-mediated endothelial dysfunction.

Breviscapine is a flavonoid extract from the traditional Chinese herb *Erigeron breviscapus*, and scutellarin (SCU) is the main active component (≥90%). Owing to its multiple pharmacological activities, including anti-inflammatory, antioxidative, anti-apoptotic, vasorelaxant, antiplatelet, and anticoagulation effects, breviscapine is commonly used to treat cerebrovascular disease, diabetic complications, and hyperlipidemia (Wang and Ma, 2018). Multiple studies have demonstrated that the concurrent use of SCU during reperfusion therapy can protect against additional myocardial injury and effectively prevent cardiac I/R injury (Pan et al., 2011; Wang et al., 2016). However, it is not yet clear whether SCU exerts its cardioprotective effects by inhibiting I/R-mediated endothelial dysfunction, and the underlying mechanisms remain unclear.

The present study was designed to determine the effects of SCU on I/R-mediated endothelial dysfunction and the underlying mechanisms. We found that SCU promoted vasodilation and reperfusion; alleviated inflammation, myocardial membrane damage, and myocardial infarction; and improved cardiac function in rats with coronary ligation-induced I/R injury. Furthermore, SCU improved cell viability and reduced cell membrane damage, ROS accumulation, and inflammation in endothelial cells after oxygen-glucose deprivation/oxygen-glucose resupply (OGD/OGR) treatment. Mechanistically, our data indicate that SCU can resist endothelial dysfunction by increasing lysosomal flow and autophagic flux, the effects of which are mediated by the direct upregulation of cathepsin D (CTSD) levels and its activity.

## 2 Materials and methods

### 2.1 Experimental animals and scutellarin administration

The 8-week-old adult male Sprague Dawley (SD) rats used in this study were housed in the Animal Center of The Second Affiliated Hospital of Guangzhou University of Chinese Medicine, at a temperature of 24°C ± 2°C with 12-hour light/dark cycles and given fresh diet and sterile water daily. Animal testing was approved by the Animal Care and Use Committee of the Second Affiliated Hospital of Guangzhou University of Chinese Medicine. All procedures were conducted in compliance with the Guide for the Care and Use of Laboratory Animals prepared by the National Academy of Sciences and published by the National Institutes of Health.

The clinically recommended dosage for breviscapus is commonly 50 mg/60 kg/day (containing SCU ≥90%). Based on the dose conversion using the standard animal weight, the human-to-rat conversion factor is 6.17. Therefore, the SCU dosage for rats in this study was calculated as follows: 50/60% × 6.17% × 90% = 4.6. Consequently, the SCU dosages were set at 5 mg/kg/day and 10 mg/kg/day. Additionally, TH is used as a positive control drug. After 1 week of adaptive feeding, a total of 48 male SD rats were randomly divided into six groups, with 8 rats per group: Sham, I/R, I/R plus TH (5 mg/kg/day)(I/R + TH), I/R plus low-dose SCU (5 mg/kg/day)(I/R + SCU L), I/R plus high-dose SCU (10 mg/kg/day)(I/R + SCU H), I/R plus SCU (10 mg/kg/day), and pepstatin A (P.A, 15 mg/kg/every other day)(IR + SCU + P.A). Rats in the I/R + SCU L, I/R + SCU H and IR + SCU + P.A groups were administered SCU (IUPAC Name: (2S,3S,4S,5R,6S)-6-[5,6-dihydroxy-2-(4-hydroxyphenyl)-4-oxochromen-7-yl]oxy-3,4,5-trihydroxyoxane-2-carboxylic acid; BD110078, purity: 97%, Bidepharm) by intraperitoneal injection for 2 weeks. Rats in the I/R + TH group were intraperitoneally injected with TH (T820041, Macklin) for 2 weeks. Rats in the IR + SCU + P.A group were intraperitoneally injected with P.A (A2571, APExBIO) every other day. All rats started administration 2 weeks before modeling, and the last dose was given half an hour before modeling. The rats in the Sham and I/R groups were intraperitoneally administered saline.

### 2.2 Establishment of an I/R injury model *in vivo*

The cardiac I/R injury model (Xie et al., 2021) was established after 2 weeks of SCU administration. Rats were anesthetized with 2% pentobarbital sodium (0.2 mL/100 g) and placed supine on a heating pad (37°C). They were intubated (16 G stump needle) and ventilated with room air using an Inspira Pressure Controlled Ventilator (Harvard Apparatus). Following left thoracotomy between the fourth and fifth ribs, the left anterior descending (LAD) coronary artery was ligated with a 7–0 Prolene suture. Regional ischemia was confirmed by observing discoloration of the myocardium distal to the occlusion site. After 30 min of ischemia, the ligation was then released. Rats in the Sham group were subjected to a sham operation (without coronary artery ligation). Follow-up experiments were performed 24 h after reperfusion. In our study, pentobarbital sodium was used as the sole anesthetic agent. No additional analgesics were administered, as the experimental procedures were designed to minimize discomfort to the animals. This approach was also taken to ensure that the results were not influenced by potential side effects of analgesic drugs.

### 2.3 Echocardiography

Echocardiographic recordings were performed using a Vevo 2100 system (Visual Sonics). Left ventricle (LV) end-diastolic and end-systolic dimensions were derived from M-mode images. All measurements were made at the level of the papillary muscles according to standard protocols. The M-mode images were analyzed using Vevo 3.1.0 software (version 1.5.0). The LV end-diastolic volume (LVEDV), stroke volume (SV), LV ejection fraction% (LVEF%), and LV fractional shortening% (LVFS%) were used to assess cardiac function in I/R rats.

### 2.4 Evans blue/triphenyltetrazolium chloride (TTC) double staining

Evans blue/TTC double staining was used to calculate the percentage of at-risk and infarction areas. Briefly, the previous occlusion of the LAD coronary artery was retied under anesthetic, and the aorta abdominalis was perfused with a 0.5% solution of Evans blue dye (E2129, Sigma) in normal saline (5 mL over 3 min). Utilizing this method, the ischemic myocardium of the LV (area at risk) was identified by the absence of blue dye, whereas the remaining LV was stained dark blue.

The heart was rapidly extracted and placed in −80°C freezer for 1 h. Subsequently, the frozen heart tissue was cut into 1.5 mm thick cross-sections, and then stained with a 1% solution of TTC (T8877, Sigma) in phosphate buffer saline (pH 7.4) at 37°C for 15 min. The heart slices were fixed in 10% neutralbuffered formaldehyde and photographed after 24 h. With this double staining, the nonischemic portion of the LV was stained dark blue, the viable tissue within the region at risk was stained red, and the infarcted tissue was white. Images of the samples were analyzed using ImageJ software. The risk area was calculated and expressed as a percentage: (red-stained area + white-stained area)/cross-sectional area × 100%. The infarction area was calculated as follows and expressed as a percentage: white stained area/(red-stained area + white-stained area) × 100%.

### 2.5 Measurement of serum cardiac troponin T (cTNT) and cardiac enzymes

After opening the abdomen and exposing the abdominal aorta, a disposable blood lancet was inserted into the abdominal aorta and 8–10 mL of blood was collected in a vacuum blood collection tube without an anticoagulant. The blood was centrifuged at room temperature at 3,500 rpm for 10 min at room temperature, followed by timely separation of serum and storage in a −80°C freezer. The serum for the detection of cardiac enzymes were temporarily stored at 4°C, and restored to room temperature 30 min before the detection. The serum levels of cTNT, creatine kinase (CK), CK-MB, lactate dehydrogenase (LDH), and α-hydroxybutyrate dehydrogenase (α-HBDH) were measured using Roche automatic biochemical analyzer.

### 2.6 Enzyme-linked immunosorbent assay (ELISA)

The serum levels of interleukin 1 beta (IL-1β), IL-6, tumor necrosis factor alpha (TNF-α) and ET-1 were determining using an ELISA kit (IL-1β, Animaluni, LV20275; IL-6, Animaluni, LV20298; TNF-α, Animaluni, LV20497; ET-1, Animaluni, LV20153) according to the manufacturer’s protocol. Briefly, samples and standards were added to the wells and incubated at 37°C for 50 min. Then, the plate was washed and biotinized antibody was added and incubated at 37°C for 50 min. The plate was washed again and Strept Avidin Biotin Enzyme Complex (SABC) was added to each well, then incubated for 30 min at 37°C. The plate was washed and tetramethylbenzidine substrate was added. After incubation for 15 min in a dark room at 37°C, stop solution was added and the optical densities were read at 450 nm wavelength using a Varioskan™ LUX Multi-function microplate reader (Thermo Fisher). The results of IL-1β, IL-6, TNF-α and ET-1 were expressed as concentrations as pg/mL.

Serum NO levels were measured using an NO assay kit (LV200005, Animaluni) following the manufacturer’s protocol. The samples and standards were added to the wells, and the extraction solution was added to each well and incubated at room temperature for 3 min. Then, color-developing solution was added, and after 5 min of incubation at room temperature, the optical densities were read at a wavelength of 540 nm using a microplate reader. The concentration was expressed as pmol/mL.

### 2.7 Immunofluorescence (IF)

To stain rat tissue sections, the animals were anesthetized and then perfused intracardially with ice-cold saline, followed by 4% paraformaldehyde (PFA). The heart and aortic vascular tissues were dissected and post-fixed overnight at 4°C in 4% PFA. After that, the tissues were embedded in paraffin and sectioned at 4 µm intervals. For IF staining, the aortic vascular tissue sections were permeabilized with 0.5% Triton X-100 in PBS for 30 min at room temperature and blocked with 10% goat serum for 1 h at room temperature. Then, the tissue sections were incubated with primary antibodies at 4°C for overnight. The corresponding fluorescence-conjugated secondary antibodies [Alexa Fluor 488/647, Abcam, goat anti-rabbit (ab150077)/mouse (ab150115)] were then incubated at room temperature in the dark for 1 h. The tissues were then stained with DAPI. Images were acquired using a Zesis confocal microscope (40× )(LSM800, Zesis) with ZEN software. The primary antibodies used for IF staining were CTSD (66534-1-Ig; Proteintech), MAP1A/1B light chain 3B (LC3Ⅱ)(18725-1-AP; Proteintech), P62 (GB11531; Servicebio), lysosomal associated membrane protein 1 (LAMP1)(9091s; Cell Signaling Technology (CST)), and platelet endothelial cell adhesion molecule-1 (CD31)(66065-2-Ig, 28083-1-AP; Proteintech).

### 2.8 Histology and immunohistochemistry (IHC)

Hematoxylin-eosin (HE) staining of heart tissue sections was performed according to standard procedures. Images were acquired via microscopy (Leica) with Leica LAS AF software.

Aortic vascular tissue was fixed with 4% PFA, followed by paraffin embedding and sectioning for IHC staining. Briefly, the paraffin sections were routinely dewaxed and washed with PBS. Tissue antigens were retrieved by ethylene diamine tetraacetic acid (EDTA) buffer (pH 9.0) microwave antigen repairing method, and the sections were incubated with 3% goat serum for 30 min, and then incubated with primary antibodies at 4°C for overnight. The secondary antibody was then incubated at room temperature for 30 min. Visualization was accomplished using a Diaminobenzidine (DAB) Peroxidase Substrate Kit (PK1006, Proteintech). Images were acquired via Leica microscopy using Leica LAS AF software. Images were then uploaded to the ImageJ software for analysis. The observation of the images was as limited as possible to the endothelial layer, which is achieved by carefully selecting the area of interest (ROI) of the aortic section, specifically on the surface of the vascular lumen. Mean optical density (MOD) was used to reflect protein abundance and was calculated as follows: integral optical density (IOD)/area. The primary antibodies used for IHC staining were von willebrand factor (vWF)(ab6994; Abcam), vascular endothelial growth factor (VEGF)(A0280; Abclonal), CTSD (66534-1-Ig; Proteintech), LC3Ⅱ (18725-1-AP; Proteintech), P62 (GB11531; Servicebio), and LAMP1 (GB14104; Servicebio).

### 2.9 Cell culture and establishment of an I/R injury model *in vitro*

The human coronary artery endothelial cells (HCAECs) were donated by Prof. Lei Wang, the Director of the Cardiovascular Department of the Second Affiliated Hospital of Guangzhou University of Chinese Medicine. HCAECs were cultured in RPMI-1640 Medium supplemented with 10% fetal bovine serum and 1% Penicillin-Streptomycin Solution (Gibco), at 37°C in 5% CO_2_ in a humidified incubator. The passage number of HCAECs used for modeling should be less than 15. An *in vitro* I/R injury model was established using the OGD/OGR approach (Hossain et al., 2021). For OGD treatment, HCAECs were cultured in D-Hank’s medium (lacking calcium and magnesium ions) for 3 h in a three-gas incubator (hypoxic conditions were set to 94% N_2_, 1% O_2_, and 5% CO_2_) (Zhuang et al., 2023; Wang et al., 2018). After that, the plates were removed and the D-Hank’s solution in each well was discarded, RPMI-1640 complete medium was added, and the plates were incubated in a CO_2_ incubator for 24 h for subsequent experiments. HCAECs were treated with SCU (12.5 µM or 25 µM), combined with or without bafilomycin A1 (BafA1, 50 nM; S1413, Selleck) at the beginning of OGR.

### 2.10 Construction of CTSD knockdown cell model

A *CTSD* knockdown cell model was constructed using small interfering RNA (siRNA) transfection. Transfection reagents (C10511-05; China) were purchased from RiboBio, who also provided the siRNAs. Following the guidelines provided by the manufacturer, the siRNAs and transfection reagents were sequentially added to 1.5 mL EP tubes. Afterwards, an appropriate amount of culture medium was added and the mixture was added to the culture plates. The culture plates were placed in a CO_2_ incubator at 37°C for 24 h. The interference effect was verified using Western blotting. The sequence of siRNA for CTSD used is 5ʹ- AGG​GTT​CTC​TGT​CCT​ACC​T -3ʹ.

### 2.11 Cell viability assay and LDH release assay

Cell viability was determined using a Cell Counting Kit-8 (CCK-8) assay (CK04, DOJINDO) according to the manufacturer’s protocol. Following a 24-h period of OGR, fresh medium containing 10% CCK-8 was added to each well, and incubated at 37°C for 3 h. The absorbance value was measured at 480 nm using a microplate reader. The cell viability rate was calculated as follows: light absorption value of the model group-light absorption value of the blank group)/(light absorption value of the control group-light absorption value of the blank group) × 100%. No cells in the blank group and only medium supplemented with 10% CCK-8 were inclued.

Culture supernatants from each well were collected after 24 h of OGR treatment. Then, 100 μL fresh medium supplemented with 15 μL 10% Triton-X 100 was added to each well, followed by incubating in CO_2_ incubator for 45 min, and cell lysates were collected. In addition, blank groups were set, including blank group 1 (culture medium without cells) and blank group 2 (cell-free only culture medium supplemented with 15 μL 10% Triton-X 100). The Roche cobas^®^8000 modular analyzer was utilized to measure the LDH concentration. The LDH release rate was calculated as the percentage of the supernatant LDH concentration relative to the total LDH concentration.

### 2.12 Quantitative real-time reverse transcription polymerase chain reaction (qPCR)

Total RNA from HCAECs was extracted using AG RNAex Pro RNA extraction reagent [AG21102, Accurate Biotechnology (AG)], according to the manufacturer’s instructions. The RNA concentration was measured using a Nanodrop 2000C instrument (NanoDrop Technologies). RNA (1 µg) was reverse transcribed into cDNA using Evo M-MLV reverse transcription reagent premix (AG11706, AG) as instructed. SYBR Green Pro Taq HS premix qPCR reagent (AG11701, AG) was used for the qPCR analysis. A Roche 480 instrument was used for amplification. Primers used in this study were as follows: *GAPDH* (glyceraldehyde-3-phosphate dehydrogenase)(Forward, 5-GGA​GCG​AGA​TCC​CTC​CAA​AAT-3; Reverse, 5-GGC​TGT​TGT​CAT​ACT​TCT​CAT​GG-3), *ICAM-1* (intercellular adhesion molecule 1)(Forward, 5-ATG​CCC​AGA​CAT​CTG​TGT​CC-3; Reverse, 5-GGG​GTC​TCT​ATG​CCC​AAC​AA-3), *MCP-1* (monocyte chemoattractant protein-1)(Forward, 5-CAG​CCA​GAT​GCA​ATC​AAT​GCC-3; Reverse, 5-TGG​AAT​CCT​GAA​CCC​ACT​TCT-3), *IL-1β* (interleukin 1 beta)(Forward, 5-ATG​ATG​GCT​TAT​TAC​AGT​GGC​AA-3; Reverse, 5-GTC​GGA​GAT​TCG​TAG​CTG​GA-3), *CTSD* (Forward, 5-TGC​TCA​AGA​ACT​ACA​TGG​ACG​C3’; Reverse, 5-CGA​AGA​CGA​CTG​TGA​AGC​ACT-3). All the primers were purchased from Thermo Fisher Scientific. Data were normalized to GAPDH, and mRNA abundance was calculated using the 2^−ΔΔCT^ method (Livak and Schmittgen, 2001).

### 2.13 ROS detection

ROS were detected using dichloro-dihydro-fluorescein diacetate (DCFH-DA) probe (S0033s; Beyotime) staining, followed by observation under a fluorescence microscope and quantification by flow cytometry. For fluorescence microscopy observation, HCAECs were incubated in RPMI-1640 medium containing 0.1% DCFH-DA probe for 1 h. Then, the culture medium containing DCFH-DA was removed, and Hoechst dye solution (Beyotime) was added for nuclear staining. Images were acquired using a fluorescence microscope (Leica) with Leica LAS AF software. ROS production was measured using flow cytometry. HCAECs in 6-well plates were harvested, transferred to labeled tubes, and centrifuged at 100 × g for 3 min. The DCFH-DA probe (at a dilution of 1:1000) was added and incubated for 20 min in darkroom. After washing with RPMI-1640 medium, 600 μL of Hank’s balanced salt solution was added. Fluorescence was detected using an Accuri C6 flow cytometer (BD Biosciences).

### 2.14 Western blot

Proteins were extracted from HCAECs using a cell lysis buffer [radioimmunoprecipitation assay (RIPA) containing 1% phenylmethylsulfonyl fluoride (PMSF) and 1% protease inhibitor cocktail (PIC)], and quantified using a BCA protein quantitation kit (FD2001, FDbio). Proteins were separated by sodium dodecyl sulfate-polyacrylamide gel electrophoresis and then transferred onto a polyvinylidene fluoride (PVDF) membrane (Roche). After being blocked at room temperature for 1 h in TBST (Tris-buffered saline containing 0.1% Tween 20) containing 5% skim milk, the membranes were incubated with primary antibodies at 4°C for overnight and followed by incubation with horseradish peroxide (HRP)-conjugated secondary antibody for 1 h at room temperature. After washing with TBST buffer, the membranes were developed using an FDbio-Dura ECL kit (FD8020, FDbio) in a Chemiluminescent Imaging System (Tanon). Band intensities were quantified using Photoshop software. Antibodies used in this experiment were as follows: MAP1A/1B light chain 3 (LC3)(3868s; CST), autophagy-related protein 5 (Atg5)(12994s; CST), autophagy-related protein 12 (Atg12)(4180s; CST), Beclin-1 (3495s; CST), P62 (39749s; CST), CTSD (A19680; Abclonal), LAMP1 (9091s; CST), ubiquitin (3933s; CST), beta-actin (ACTB)(AC004; Abclonal), and HRP-conjugated secondary antibodies [Anti-rabbit IgG (7074s; CST); Anti-mouse IgG (7076s; CST)].

### 2.15 Cycloheximide (CHX) chase analysis

To determine CTSD protein degradation, HCAECs were cultured in media with cycloheximide (50 μg/mL) followed by OGR for 24 h, and cells were collected at different time points on ice. CTSD protein levels for each sample was measured by Western blot and quantified using normalized ratio of CTSD to ACTB.

### 2.16 CTSD activity assay

CTSD activity was assessed using a CTSD activity assay kit (K143; BioVision) following the manufacturer’s instructions. The cell lysates were obtained with CD Cell Lysis Buffer and followed by centrifugation at 14,000 × g, for 10 min at 4°C. Next, the supernatants were transferred to 1.5 mL labeled tubes, and the amount of protein was quantified using a Nanodrop 2000c instrument. Following that, equal amounts of protein (5 ng) were added to each well of a 96-well plate, along with 52 μL of Master Assay mixture (containing 50 μL of CD Reaction Buffer and 2 μL CD Substrate). The plate was then incubated at 37°C for 2 h. Fluorescence was measured at excitation and emission wavelengths of 328 and 460 nm, respectively. Data are presented as the fold-change relative to the control.

### 2.17 LysoTracker and LysoSensor double staining

Following 24 h OGR treatment, HCAECs were incubated with LysoTracker (1 μM; 40739 ES, Yeasen Biotechnology) and LysoSensor (1 μM; 40767ES50, Yeasen Biotechnology) in RPMI-1640 medium for 1 h at 37°C. The medium was then discarded and the cells were washed in pre-warmed PBS (×3) to remove the unbound probes. Live-imaging was performed using a Leica confocal microscope (63×)(TCS SPE-Ⅱ, Leica). Images were captured from six random fields for each group using the same setting parameters (the time taken to image each field was controlled to within 5 s). Leica software was used to determine the Lysosensor intensity in the LysoTracker positive area of each cell, and the results were documented in Excel.

### 2.18 Statistical analysis

All data are displayed as the mean ± standard deviation (SD). Statistical analyses were conducted using SPSS software (version 22.0), GraphPad Prism 8.0 was used for data visualization. Adobe Illustrator CC software was employed for figure creation. Comparisons between the two groups were performed using a two-tailed unpaired Student’s-test. Comparison of multiple groups were compared using one-way analysis of variance (ANOVA) followed by the Bonferroni test. *P* < 0.05 was considered statistically significant.

## 3 Results

### 3.1 Scutellarin attenuated I/R-induced myocardial infarction and improved cardiac function

**FIGURE 1:**
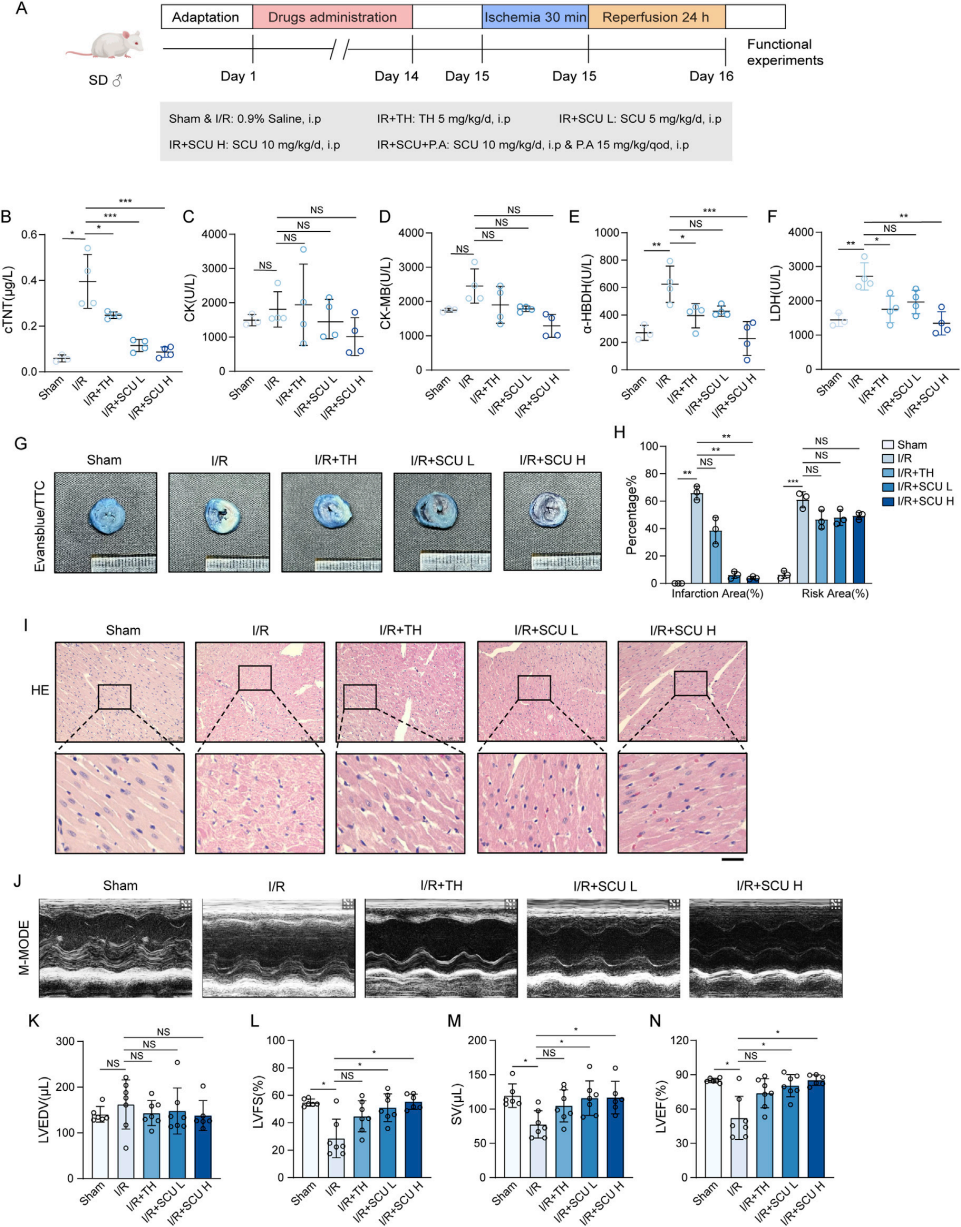
Scutellarin attenuated I/R-induced myocardial infarction and rescued cardiac function. **(A)** Schematic diagram of the point in drugs administration. **(B–F)** Quantification of serum cTNT **(B)**, CK **(C)**, CK-MB **(D)**, LDH **(E)** and a-HBDH **(F)** levels in sham, I/R, I/R+TH, I/R+SCU L and I/R+SCU H groups (n = 4). **(G)** Representative photos of evans blue/TTC staining and **(H)** quantification of risk area and infarction area sizes (n = 3). **(I)** Representative micrographs of HE staining. Scale bars, 100 μm. **(J)** Representative acquisition of M-mode images. **(K–N)** Quantification of LVEDV (μL) **(K)**, LVFS(%) **(L)**, SV (μL) **(M)** and LVEF (%) **(N)** in sham, I/R, I/R+TH, I/R+SCU L and I/R+SCU H groups (n = 7 for each group). Data shown are means ± SD; _NS_ *P*> 0.05, *P< 0.05, **P< 0.01 and ***P< 0.001 vs. the sham group or I/R group. I/R, ischemia/reperfusion; TH, tirofiban hydrochloride; SCU L, scutellarin low; SCU H, scutellarin high; cTNT, cardiac troponin T; CK, creatine kinase; CK-MB, creatine kinase-MB; LDH, lactate dehydrogenase; a-HBDH, alpha hydroxybutyrate dehydrogenase; TTC, triphenyltetrazolium chloride; HE, Hematoxylin-eosin; LVEDV, left ventricular end systolic volume; LVFS, left ventricular fraction shortening; SV, stroke volume; LVEF, left ventricular ejection fraction.

To explore the pharmacological effects of SCU on the cardiac I/R injury, rats were subjected to coronary artery ligation followed by reperfusion on rats to induce I/R injury (The flow chart is shown in Figure 1A). Elevated levels of serum myocardial enzymes in serum is a pathological feature of cardiac I/R injury. Our results showed (Figures 1B–F; Table 1) that coronary artery ligation followed by reperfusion resulted in enhanced serum myocardial enzymes (cTNT, CK, CK-MB, LDH and α-HBDH) in the I/R group. However, the serum levels of myocardial enzymes were notably reduced in the I/R + SCU L, I/R + SCU H and I/R + TH groups, with even lower levels in the I/R + SCU group than in the I/R + TH group, indicating that SCU can more effectively and stably ameliorate myocardial damage induced by I/R. The area of myocardial infarction is an important marker that reflects the severity of I/R injury, and both Evans blue/TTC double staining and HE staining can be used to evaluate myocardial infarction changes. As shown in Figures 1G, H, The I/R group demonstrated a noticeable increase in the risk and infarction areas via Evans blue/TTC staining, whereas SCU-pretreated rats displayed a remarkable reduction in the infarction areas. In addition, HE staining showed (Figure 1I) that the myocardial arrangement was disordered and there was extensive hemorrhage along with myocardial fiber necrosis, loss of nuclei, and cross striations in the I/R group, which is consistent with the microscopic view of a recent myocardial infarction. However, these phenomena were reversed by SCU pretreatment, suggesting that SCU can improve the effects of reperfusion therapy and inhibit myocardial infarction. To evaluate the functional implications of the myocardial infarction size, echocardiography was performed to assess myocardial contractility. Consistent with the increased infarction area, I/R rats displayed a reduction in contractile performance, quantified as lower LVFS%, SV, and LVEF%, compared to the Sham group, while SCU-pretreated rats exhibited a significant improvement in contractile performance, which was consistent with the decreased risk area and infarction size (Figures 1J–N; Table 3). These findings suggest that SCU protects rats from cardiac I/R injury.

**TABLE 1:** Serum cardiac troponin T (cTNT) and cardiac enzymes results.

Indicator	Sham (n = 3)	I/R (n = 4)	I/R + TH (n = 4)	I/R + SCU L (n = 4)	I/R + SCU H (n = 4)	I/R + SCU + P.A (n = 3)
cTNT (μg/L)	0.1 ± 0.0	0.4 ± 0.1^*^	0.2 ± 0.0^†^	0.1 ± 0.0^†††^	0.1 ± 0.0^†††^	0.3 ± 0.0^‡^
CK (U/L)	1,493.3 ± 94.8	1807.0 ± 257.0^NS^	1942.0 ± 593.0^NS^	1,527.3 ± 287.9^NS^	1,012.0 ± 277.3^NS^	2021.3 ± 312.7^NS^
CK-MB (U/L)	1749.2 ± 32.1	2452.4 ± 249.7^NS^	1903.3 ± 269.6^NS^	1786.5 ± 38.7^NS^	1,290.7 ± 165.5^NS^	2502.6 ± 234.6^‡^
α-HBDH (U/L)	270.0 ± 31.6	624.5 ± 66.3^**^	395.0 ± 44.2^†^	427.8 ± 18.7^NS^	228.5 ± 62.0^†††^	799.7 ± 151.0^‡‡^
LDH (U/L)	1,444.0 ± 111.5	2713.5 ± 198.8^**^	1750.8 ± 193.4^†^	1965.8 ± 170.7^NS^	1,346.0 ± 169.7^††^	3,126.3 ± 315.5^‡‡^

Note. Data shown are means ± SD. ^NS^ *P > *0.05, ^*^ *P* < 0.05, ^**^ *P* < 0.01 and ^***^ *P* < 0.001 vs. the Sham group; ^NS^ *P > *0.05, ^†^ *P* < 0.05, ^††^ *P* < 0.01 and ^†††^ *P* < 0.001 vs. the I/R group; ^NS^ *P > *0.05, ^‡^ *P* < 0.05, ^‡‡^ *P* < 0.01 and ^‡‡‡^ *P* < 0.001 vs. the I/R + SCU H group. I/R, ischemia/reperfusion; TH, tirofiban hydrochloride; SCU L, scutellarin low; SCU H, scutellarin high; P.A, pepstatin A; cTNT, cardiac troponin T; CK, creatine kinase; CK-MB, creatine kinase-MB; LDH, lactate dehydrogenase; a-HBDH, alpha hydroxybutyrate dehydrogenase.

### 3.2 Scutellarin attenuated I/R-mediated endothelial dysfunction *in vivo* and *in vitro*

**FIGURE 2:**
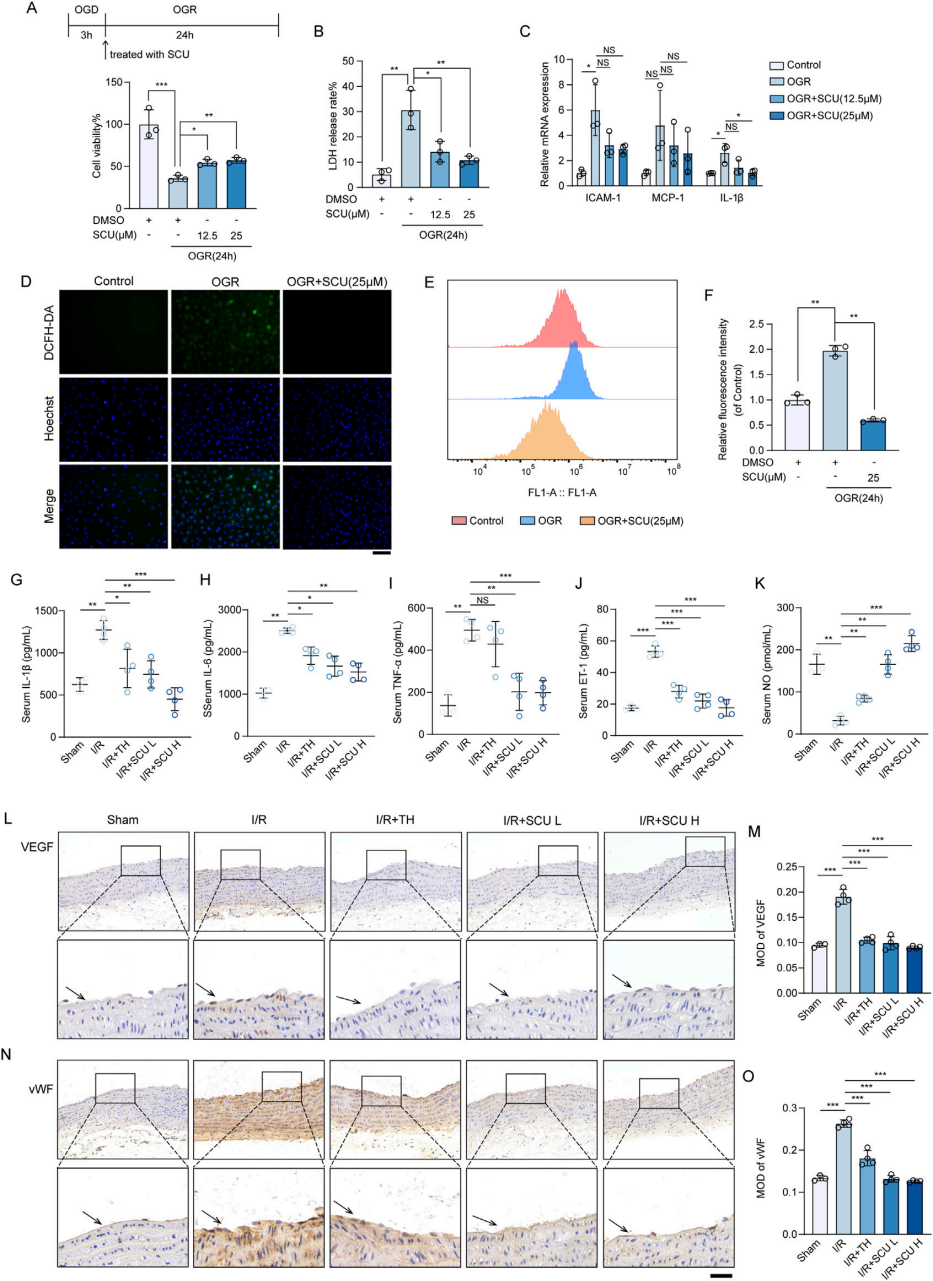
Scutellarin attenuated I/R-induced endothelial dysfunction *in vivo* and in vitro. **(A)** Schematic diagram of the point in time design (Top). Cell viability in control, OGR+DMSO, OGR+SCU HCAECs. Results are expressed as a percentage of the control group (n = 3). **(B)** Assessment of the LDH release rate (n = 3). **(C)** Quantification of ICAM-1, MCP-1, and IL-1β mRNA levels in HCAECs, normalized to GAPDH expression (n = 3). **(D)** Representative images showing DCFH-DA- and Hoechst 33258-labeled cells in control, OGR(24 h)+DMSO, OGR (24 h)+SCU groups. Scale bars, 100 μm. **(E)** Histogram and **(F)** quantification of fluorescence intensity of ROS (n = 3). **(G-K)** Quantification of serum IL-1β **(G)**, IL-6 **(H)**, TNF-α **(I)**, ET-1 **(J)** and NO **(K)** levels in sham, I/R, I/R+TH, I/R+SCU L and I/R+SCU H groups (n = 4). **(L)** Representative IHC images and **(M)** quantification of VEGF in the aortic vascular tissues of sham, I/R, I/R+SCU L and I/R+SCU H rats (n = 4). Scale bars, 100 μm. **(N)** Representative IHC images and **(O)** quantification of vWF in the aortic vascular tissues of sham, I/R, I/R+SCU L and I/R+SCU H rats (n = 4). Scale bars, 100 μm. Data shown are means ± SD; _NS_ *P* > 0.05, *P < 0.05, **P < 0.01 and ***P < 0.001 vs. the indicated group. OGD, oxygen-glucose deprivation; OGR, oxygen-glucose resupply; SCU, scutellarin; DMSO, dimethylsulfoxide; LDH, lactate dehydrogenase; ICAM-1, intercellular adhesion molecule 1; MCP-1, monocyte chemoattractant protein 1; IL-1β, interleukin 1 beta; GAPDH, glyceraldehyde-3-phosphate dehydrogenase; DCFH-DA, dichloro-dihydro-fluorescein diacetate; ROS, reactive oxygen species; I/R, ischemia/reperfusion; TH, tirofiban hydrochloride; SCU L, scutellarin low; SCU H, scutellarin high; IL-6, interleukin 6; TNF-α, tumor necrosis factor-alpha; ET-1, Endothelin 1; NO, nitric oxide; VEGF, vascular endothelial growth factor; vWF, von willebrand factor.

**FIGURE 3:**
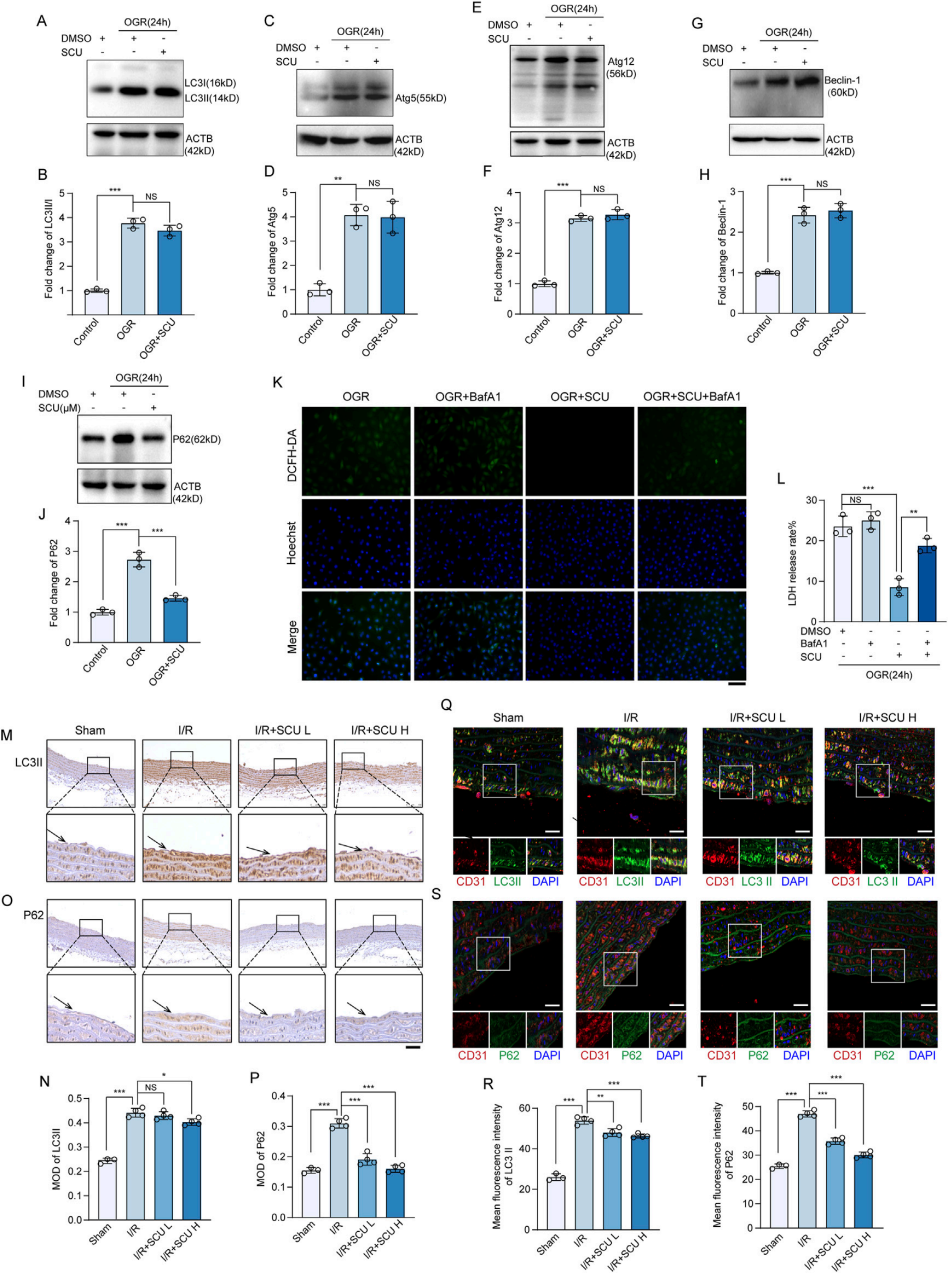
Scutellarin has no effect on autophagy initiation but can rescue the impaired autophagy flux induced by I/R. **(A)** Western blot and **(B)** quantification of LC3II/I levels in HCAECs. **(C)** Western blot and **(D)** quantification of Atg5 levels. **(E)** Western blot and **(F)** quantification of Atg12 levels. **(G)** Western blot and **(H)** quantification of Beclin-1 levels. **(I)** Western blot and **(J)** quantification of P62 levels. LC3 II/I, Atg5, Atg12, Beclin-1 and P62 levels were normalized to ACTB (n = 3 for each group). **(K)** Representative images showing DCFH-DA- and Hoechst 33258-labeled cells in OGR+DMSO, OGR+BafA1, OGR+SCU, OGR+SCU+BafA1 groups. Scale bars, 100 μm. **(L)** Assessment of the rate of LDH release (n = 3). **(M)** Representative IHC images and **(N)** quantification of LC3II in the aortic vascular tissues of sham, I/R, I/R+SCU L and I/R+SCU H rats (n = 4). Scale bars, 100 μm. **(O)** Representative IHC images and **(P)** quantification of P62 in the aortic vascular tissues of sham, I/R, I/R+SCU L and I/R+SCU H rats (n = 4). Scale bars, 100 μm. **(Q)** Representative IF images showing staining of vascular endothelial cells with CD31 (Red) and LC3II (Green) in sham, I/R, I/R+SCU L and I/R+SCU H rats. Scale bars, 20 μm. **(R)** Quantification of LC3II levels in CD31-stained positive cells (n = 4). **(S)** Representative IF images showing staining of vascular endothelial cells with CD31 (Red) and P62 (Green) in sham, I/R, I/R+SCU L and I/R+SCU H rats. Scale bars, 20 μm. **(T)** Quantification of P62 levels in CD31-stained positive cells (n = 4). Data shown are means ± SD; _NS_ *P* > 0.05, *P < 0.05, **P < 0.01 and ***P < 0.001 vs. the indicated group. OGR, oxygen-glucose resupply; SCU, scutellarin; DMSO, dimethylsulfoxide; BafA1, bafilomycin A1; LC3II/I, MAP1A/1B light chain 3B/A; Atg5/12, autophagy-related protein 5/12; ACTB, beta-actin; LDH, lactate dehydrogenase; DCFH-DA, dichloro-dihydro-fluorescein diacetate; ROS, reactive oxygen species; I/R, ischemia/reperfusion; SCU L, scutellarin low; SCU H, scutellarin high; CD31, platelet endothelial cell adhesion molecule-1; MOD, mean of optical density.

Because we found that SCU pretreatment could effectively improve the therapeutic effect of reperfusion, suggesting that SCU pretreatment is beneficial for microcirculation and blood reflow, we speculated that SCU might alleviate I/R-mediated endothelial dysfunction. We used an OGD/OGR-induced endothelial cell injury model to further assess the potential benefits of SCU on the endothelial dysfunction. As shown in Figures 2A, B, the OGD/OGR group had decreased cell viability compared to the control group, but exhibited a higher LDH release rate than the control group. However, the cell viability was increased and the LDH release rate was remarkably decreased in the endothelial cells after OGD/OGR treatment with SCU, suggesting that SCU ameliorated the endothelial cell injury induced by OGD/OGR. QPCR assays showed (Figure 2C) that the mRNA levels of inflammatory cytokines (*IL-1β*) and adhesion molecules (*ICAM-1* and *MCP-1*) associated with endothelial dysfunction were elevated to varying degrees in OGD/OGR treated cells while SCU reversed these phenomena. Moreover, previous studies have demonstrated that the excessive production of ROS triggered by I/R is the primary factor in endothelial injury, and that the accumulation of ROS can can serve as an indicator of I/R-mediated endothelial dysfunction. We performed two different methods to detect intracellular ROS levels. DCFH-DA fluorescent probe staining (Figure 2D) revealed that ROS aggregation in the endothelial cells after OGD/OGR treatment was inhibited by SCU. Similar results were obtained using flow cytometry to detect ROS (Figures 2E, F). We consistently observed comparable protective effects of SCU on endothelial cells in an *in vivo* I/R injury model. Our results showed (Figures 2G–K; Table 2) that the serum levels of inflammatory cytokines (IL-1β, IL-6 and TNF-α) and vasoconstriction related active peptide (ET-1) were noticeably increased in I/R rats, while serum levels of NO related to vasodilation were reduced, suggesting alterations in endothelial function. To provide more direct evidence of endothelial dysfunction, we included IHC staining for VEGF and vWF in aortic vascular tissues. As shown in Figures 3L–O, the VEGF and vWF levels were increased in the aortic vascular tissue of I/R rats, compared to Sham rats. Changes in these factors in the I/R group indicated endothelial dysfunction among these rats. However, pretreatment with SCU reversed this phenomenon in a concentration-dependent manner, which was mainly manifested as the reduction of serum inflammatory factors (IL-1β, IL-6 and TNF-α) and ET-1 levels, the elevation of NO levels, and the decrease in vWF and VEGF levels (as shown in Figures 3G–O; Table 2). Notably, our results showed that SCU pretreatment could regulate the changes of endothelial dysfunction markers more significantly than TH pretreatment, indicating that SCU pretreatment may has a more stable endothelial protective effect. Collectively, the aforementioned findings demonstrated that SCU has the ability to safeguard endothelial cells from I/R injury.

**TABLE 2:** Enzyme-linked immunosorbent assay (ELISA) results.

Indicator	Sham (n = 3)	I/R (n = 4)	I/R + TH (n = 4)	I/R + SCU L (n = 4)	I/R + SCU H (n = 4)	I/R + SCU + P.A (n = 3)
IL-1β (pg/mL)	624.4 ± 81.4	1,271.8 ± 111.7^**^	815.5 ± 229.0^†^	745.2 ± 161.9^††^	450.7 ± 135.3^†††^	1,689.4 ± 321.2^‡‡‡^
IL-6 (pg/mL)	1,023.6 ± 116.6	2506.2 ± 66.2^**^	1912.9 ± 206.1^†^	1,663.4 ± 239.0^†^	1,522.6 ± 210.5^††^	2821.2 ± 320.8^‡^
TNF-α (pg/mL)	137.2 ± 50.6	494.8 ± 51.0^**^	429.0 ± 107.7^NS^	202.2 ± 88.1^††^	198.1 ± 57.8^†††^	707.1 ± 34.7^‡‡‡^
ET-1 (pg/mL)	17.5 ± 1.7	53.3 ± 3.7^***^	27.9 ± 4.0^†††^	22.0 ± 4.4^†††^	17.6 ± 5.3^†††^	58.4 ± 4.5^‡‡‡^
NO (pmol/mL)	165.8 ± 24.1	31.9 ± 10.3^**^	84.3 ± 7.9^††^	165.5 ± 22.9^††^	214.8 ± 18.8^†††^	75.4 ± 13.2^‡‡‡^

Note. Data shown are means ± SD. ^*^ *P* < 0.05, ^**^ *P* < 0.01 and ^***^ *P* < 0.001 vs. the Sham group; ^NS^ *P > *0.05, ^†^ *P* < 0.05, ^††^ *P* < 0.01 and ^†††^ *P* < 0.001 vs. the I/R group; ^‡^ *P* < 0.05, ^‡‡^ *P* < 0.01 and ^‡‡‡^ *P* < 0.001 vs. the I/R + SCU H group. I/R, ischemia/reperfusion; TH, tirofiban hydrochloride; SCU L, scutellarin low; SCU H, scutellarin high; P.A, pepstatin A; IL-1β, interleukin 1 beta; IL-6, interleukin 6; TNF-α, tumor necrosis factor-alpha; ET-1, Endothelin 1; NO, nitric oxide.

### 3.3 Scutellarin has no effect on autophagy initiation but can rescue the impaired autophagy flux induced by I/R

To investigate the underlying mechanism by which SCU attenuates I/R-mediated endothelial dysfunction, we collected cell lysates from endothelial cells exposed to OGD/OGR to determine the expression levels of autophagy-related proteins. Our data showed (Figures 3A–H) that LC3Ⅱ, Atg5, Atg12, and Beclin-1 were highly expressed in OGD/OGR treated endothelial cells, suggesting that OGD/OGR initiated autophagy in endothelial cells. However, we also found that the levels of P62, which is selectively degraded by the autophagy (Bae et al., 2013), were notably elevated, similar to other autophagy-related proteins (Figures 3I, J), revealing that autophagic degradation was inhibited while autophagic initiation was intact. Interestingly, all proteins except P62 failed to show a further increase while P62 was remarkably decreased after treatment with SCU (Figures 3A–J). A similar expression pattern was observed in the vascular endothelial tissue. As shown in Figures 3M–P, IHC staining data showed that LC3Ⅱ and P62 levels were significantly increased in the aortic vascular tissues of I/R rats. For IF staining of vascular tissue sections, we used anti-CD31 antibody to mark endothelial cells, and then observed CTSD levels in CD31-stained positive cells. After 24 h of reperfusion, we observed LC3Ⅱ and P62 were highly expressed in the aortic endothelium of I/R rats (Figures 3Q–T), whereas pretreatment with SCU could significantly reduced the levels of P62 (Figures 3M–T). Together, these results showed that SCU treatment had no effect on autophagic initiation but could rescue the impaired autophagic flux induced by I/R.

Our prior work has shown that disrupted autophagic flux can be detrimental and may contribute to OGD/OGR-induced endothelial dysfunction. Given that SCU can restore the compromised autophagic process, it is possible that SCU can shield endothelial cells from I/R injury by enhancing autophagic activity. To verify this hypothesis, we evaluated the pharmacological effects of SCU on endothelial cells exposed to OGD/OGR in the presence of BafA1, which inhibits autolysosomal degradation. We found that the effects of SCU on reducing LDH release and inhibiting ROS production were reversed by BafA1, indicating that promoting the autophagic flux was one of the ways in which SCU protected endothelial cells (Figures 3K, L).

### 3.4 Scutellarin attenuated I/R-induced lysosomal dysfunction

**FIGURE 4:**
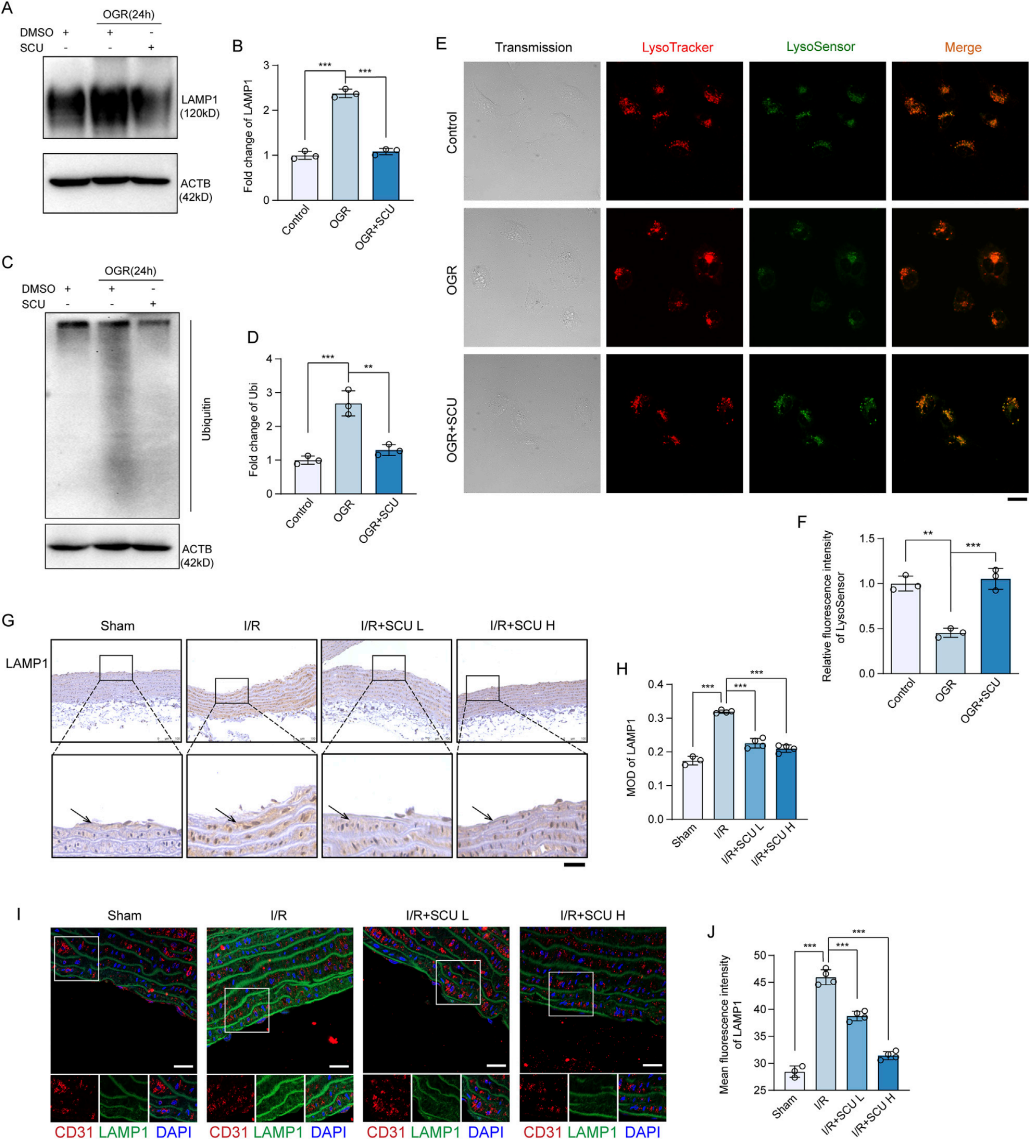
Scutellarin attenuated I/R-induced lysosomal dysfunction. **(A)** Representative western blot and **(B)** quantification of LAMP1 levels in control, OGR and OGR+SCU HCAECs. **(C)** Western blot and **(D)** quantification of ubiquitinated protein levels. LAMP1 and Ubiquitin levels were normalized to the levels of ACTB (n = 3 for each group). **(E)** Confocal images showing staining of lysosomes with LysoTracker (red) and LysoSensor (Green) in control, OGR and OGR+SCU HCAECs. Scale bars, 20 μm. **(F)** Quantification of LysoSensor fluorescence intensity (n = 3). **(G)** Representative IHC images and **(H)** quantification of LAMP1 in the aortic vascular tissues of sham, I/R, I/R+SCU L and I/R+SCU H rats (n = 4). Scale bars, 100 μm. **(I)** Representative IF images showing staining of vascular endothelial cells with CD31 (Red) and LAMP1 (Green) in sham, I/R, I/R+SCU L and I/R+SCU H rats. Scale bars, 20 μm. **(J)** Quantification of LAMP1 levels in CD31-stained positive cells (n = 4). Data shown are means ± SD; _NS_ *P* > 0.05, *P < 0.05, **P < 0.01 and ***P < 0.001 vs. the indicated group. OGR, oxygen-glucose resupply; SCU, scutellarin; DMSO, dimethylsulfoxide; LAMP1, lysosomal associated membrane protein 1; Ubi, ubiquitin; ACTB, beta-actin; I/R, ischemia/reperfusion; SCU L, scutellarin low; SCU H, scutellarin high; CD31, platelet endothelial cell adhesion molecule-1; MOD, mean of optical density.

During the autophagy process, damaged proteins and organelles are transported by autophagosomes to lysosomes for degradation (Levine and Kroemer, 2019). Since the results of our previous experiment demonstrate that SCU promotes autophagic degradation in endothelial cells, the effects of SCU on lysosomal function in endothelial cells were further investigated. We first detected the levels of LAMP1 in OGD/OGR treated endothelial cells using Western blotting. LAMP1 is a lysosomal membrane protein that is stable under normal conditions, but can accumulate during lysosomal storage diseases (Sambri et al., 2017). OGD/OGR treatment induced the accumulation of LAMP1 in endothelial cells (Figures 4A, B). LAMP1 levels in aortic endothelium also exhibited an accumulation pattern after I/R (Figures 4G–J). Nevertheless, SCU treatment largely reversed LAMP1 accumulation in both endothelial cells exposed to OGD/OGR and in the aortic endothelium of I/R rats, revealing that SCU treatment could increase lysosomal flow. At the same time, long-lived proteins were conjugated with ubiquitins and were targeted to the lysosomes for degradation (Khosravi et al., 2020; Victoria and Zurzolo, 2017); therefore, the clearance of ubiquitinated proteins can be used as another indicator of lysosomal function. Western blot results showed a significant increase in ubiquitinated proteins in OGD/OGR-exposed endothelial cells, whereas SCU inhibited ubiquitinated protein accumulation (Figures 4C, D). Furthermore, LysoTracker DND-99 and LysoSensor DND-189 double staining was performed to assess lysosomal function. Confocal image analysis of lysosomal staining with these probes demonstrated that the fluorescence signals of LysoSensor DND-189 were decreased in LysoTracker-positive puncta in endothelial cells subjected to OGD/OGR, which was effectively reversed by SCU (Figures 4E, F), suggesting that SCU rescued impaired lysosomal acidification. These findings further indicated that lysosomal degradation was hindered under I/R conditions, but SCU rescued lysosomal function, thereby promoting impaired autophagic flux.

### 3.5 Scutellarin upregulated CTSD levels and its activity in endothelial cells

**FIGURE 5:**
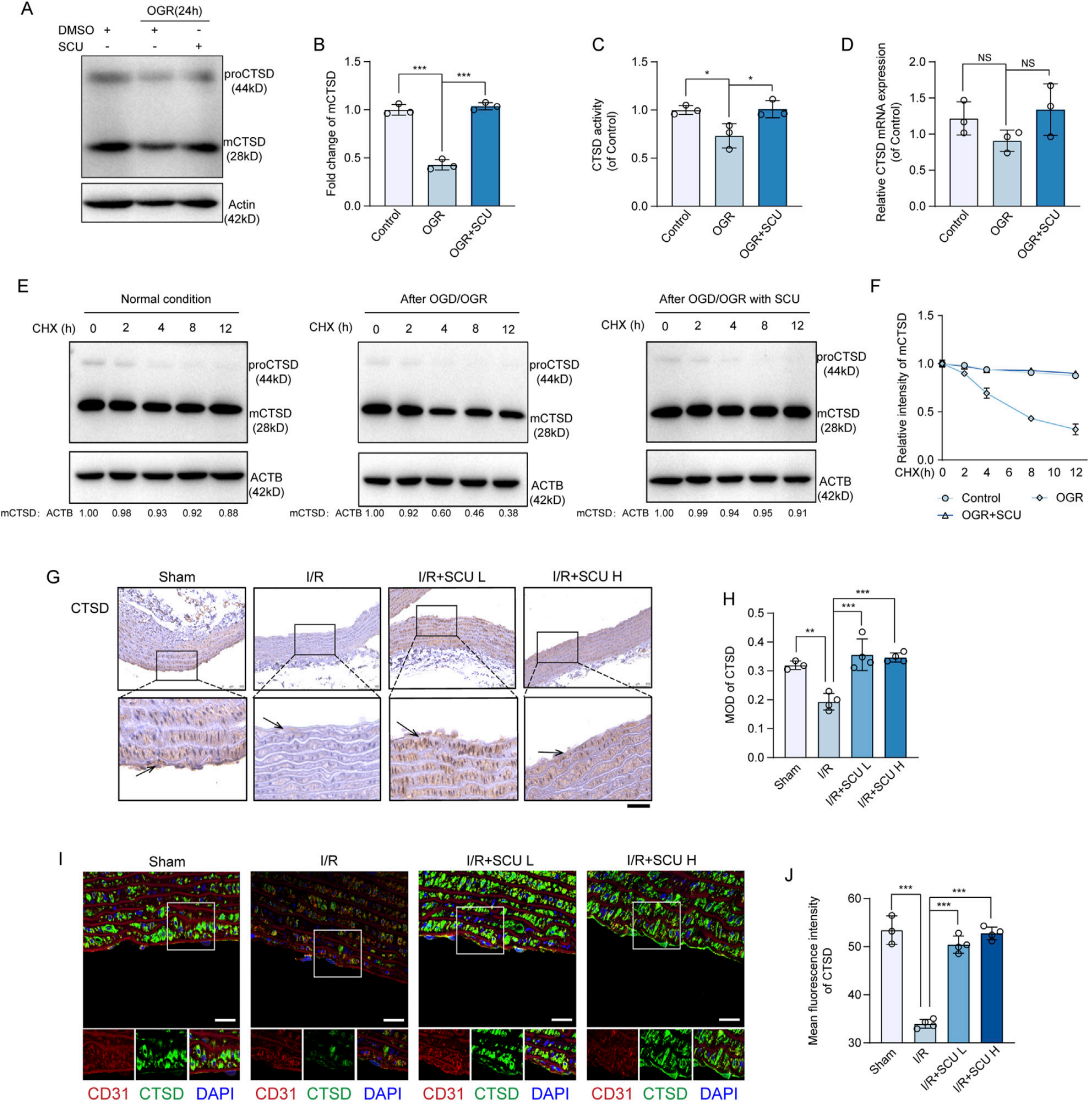
Scutellarin upregulated CTSD levels and its activity in endothelial cells. **(A)** Representative western blot and **(B)** quantification of mCTSD levels in control, OGR and OGR+SCU HCAECs. mCTSD levels were normalized to the levels of ACTB (n = 3). **(C)** Assessment of CTSD activity in control, OGR and OGR+SCU HCAECs. **(D)** Quantification of *CTSD* mRNA levels, normalized to *GAPDH* expression. n=3 for each group. **(E)** Representative western blot and **(F)** quantification of mCTSD levels in control, OGR and OGR+SCU HCAECs. HCAECs were cultured as in Figure 2A (Top) and then treated with CHX as indicated prior to western blot analysis. mCTSD levels were normalized to the levels of ACTB (n = 3). **(G)** Representative IHC images and **(H)** quantification of CTSD in the aortic vascular tissues of sham, I/R, I/R+SCU L and I/R+SCU H rats (n = 4). Scale bars, 100 μm. **(I)** Representative IF images showing staining of vascular endothelial cells with CD31 (Red) and CTSD (Green) in sham, I/R, I/R+SCU L and I/R+SCU H rats. Scale bars, 20 μm. **(J)** Quantification of LAMP1 levels in CD31-stained positive cells (n = 4). Data shown are means ± SD; _NS_ *P* > 0.05, *P< 0.05, **P < 0.01 and ***P < 0.001 vs. the indicated group. OGD, oxygen-glucose deprivation; OGR, oxygen-glucose resupply; SCU, scutellarin; DMSO, dimethylsulfoxide; CHX, Cycloheximide; mCTSD, mature cathepsin D; ACTB, beta-actin; I/R, ischemia/reperfusion; SCU L, scutellarin low; SCU H, scutellarin high; CD31, platelet endothelial cell adhesion molecule-1; MOD, mean of optical density.

Thus far, we have demonstrated that SCU rescues the lysosomal function and autophagic flux and inhibits I/R-mediated endothelial dysfunction. Next, we explored the mechanism by which SCU rescued lysosomal function and autophagic flux. Previously, we found that the maintenance of lysosomal function and autophagic flux required the involvement of CTSD and that decreased CTSD levels or enzyme activity could contribute to lysosomal dysfunction and impaired autophagic flux (Zhuang et al., 2023). Therefore, we monitored the effects of SCU on CTSD expression in endothelial cells. Our data showed that the levels of fully-mature CTSD (mCTSD) in endothelial cells exposed to OGD/OGR decreased, whereas SCU treatment reversed this trend (Figures 5A, B). However, the qPCR results showed that the mRNA levels of *CTSD* remained constant after SCU treatment (Figure 5C), indicating that SCU might affect CTSD levels via post-transcriptional regulation or degradation, rather than transcriptional regulation. To further determine whether SCU regulates CTSD degradation, we examined CTSD protein levels upon the treatment with CHX, a well-documented ribosome inhibitor, often used to block protein synthesis (Tian et al., 2021). Our time-course analysis showed that OGD/OGR treatment reduced CTSD (Figures 5E, F), indicating that protein degradation plays an important role in OGD/OGR-mediated CTSD downregulation. However, after SCU treatment, the reduction of CTSD in endothelial cells induced by OGD/OGR was inhibited (Figures 5E, F), suggesting that SCU could reduce the degradation of CTSD, which might be one of the ways in which SCU upregulates CTSD protein levels. The *in vitro* observations were further verified in the aortic vascular tissue by IHC and IF. As shown in Figures 5G, H, IHC staining data showed that CTSD levels were significantly decreased in the aortic vascular tissues of I/R rats. IF staining data showed that I/R stimulation significantly reduced CTSD levels in CD31-stained positive cells, whereas SCU pretreatment could inhibited this reduction (Figures 5I, J). Furthermore, CTSD protease activity was measured in endothelial cells exposed to OGD/OGR. The results showed that OGD/OGR decreased CTSD protease activity, whereas SCU increased CTSD protease activity (Figure 5D). These findings collectively demonstrate the positive regulation of CTSD protein levels and enzyme activity by SCU.

### 3.6 CTSD is required for SCU to rescue lysosomal function and autophagic flux

**FIGURE 6:**
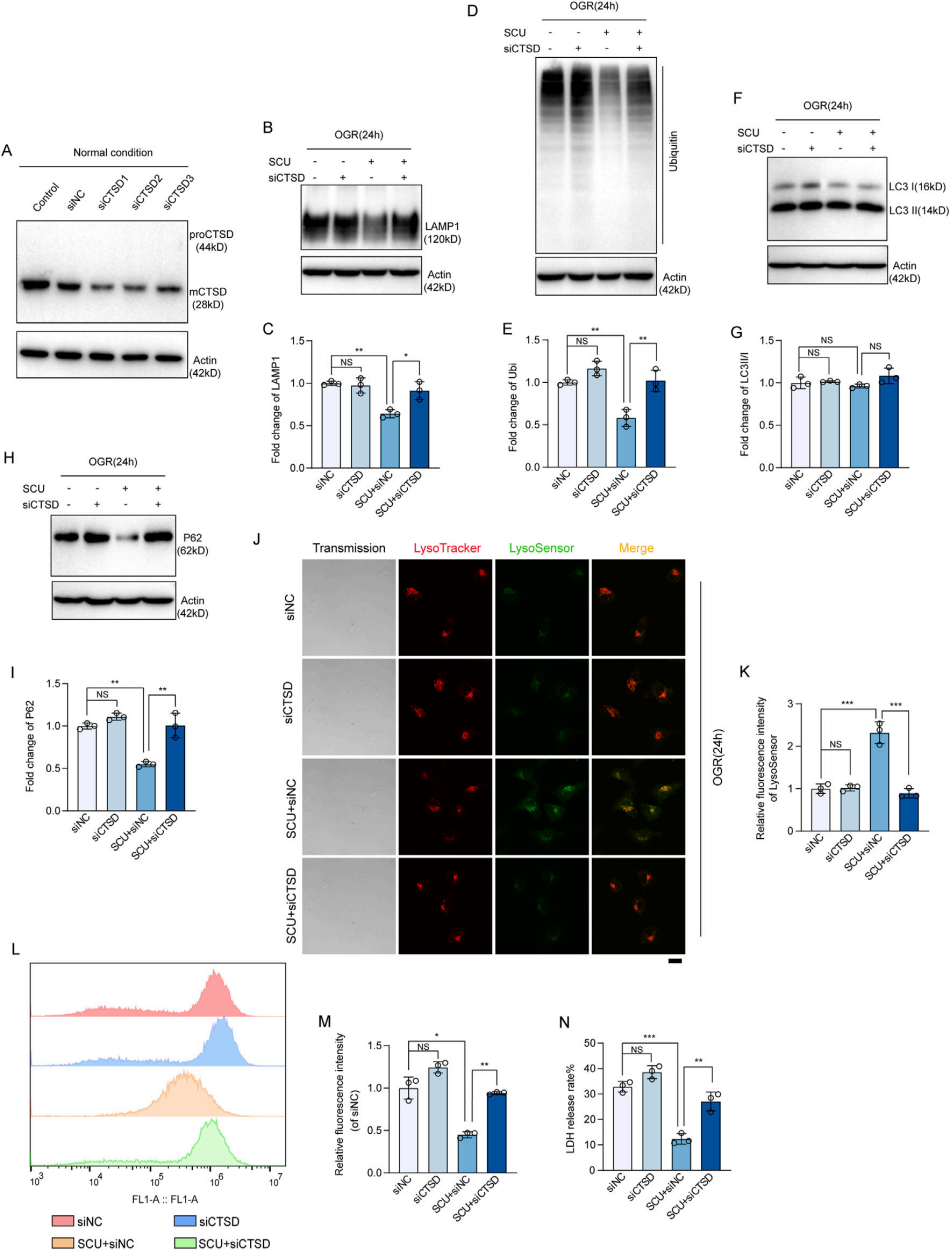
CTSD is required for SCU to rescue lysosomal function and autophagic flux. **(A)** Western blot showing the knockdown of CTSD in HCAECs using siRNA transfection. ACTB was used as a loading control. **(B)** Western blot and **(C)** quantification of LAMP1 levels in siNC, siCTSD, SCU+siNC and SCU+siCTSD HCAECs. **(D)** Western blot and **(E)** quantification of ubiquitinated proteins levels. **(F)** Western blot and **(G)** quantification of LC3II/I levels. **(H)** Western blot and **(I)** quantification of P62 levels. LAMP1, Ubiquitin, LC3II/I, and P62 levels were normalized to ACTB (n = 3 for each group). **(J)** Representative confocal images of siNC, siCTSD, SCU+siNC and SCU+siCTSD HCAECs stained with LysoTracker (Red) and LysoSensor (Green). Scale bars, 20 μm. **(K)** Quantification of LysoSensor fluorescence intensity (n = 3). **(L)** Histogram and **(M)** quantification of fluorescence intensity of ROS (n = 3). **(N)** Assessment of the LDH release rate in siNC, siCTSD, SCU+siNC and SCU+siCTSD HCAECs (n = 3). Data are presented as the mean ± SD. _NS_ *P* > 0.05, *P < 0.05, **P < 0.01 and ***P < 0.001 vs. the indicated group. OGR, oxygen-glucose resupply; SCU, scutellarin; siRNA, small interfering RNA; NC, negative control; mCTSD, mature cathepsin D; LAMP1, lysosomal associated membrane protein 1; Ubi, ubiquitin; LC3II/I, MAP1A/1B light chain 3B/A; ACTB, beta-actin.

To provide direct evidence supporting that the upregulation of CTSD levels is required for SCU to rescue lysosomal function and autophagic flux, we performed recovery experiments by conducting a *CTSD* knockdown cell line (Figure 6A). As expected, Western blot data (Figures 6B–I) showed that under OGD/OGR conditions, compared with the siNC group, the knockdown of CTSD slightly further impaired lysosomal degradation function (quantified as higher LAMP1 and ubiquitinated protein levels), and reduced autophagic flux (quantified as higher LC3Ⅱ/Ⅰ and P62 levels), but the difference between the two groups was not significant, indicating the decrease in CTSD levels caused by OGD/OGR treatment is sufficient to significantly disrupt lysosomal degradation function and autophagic flux in endothelial cells. But as shown in Figures 6B–I, we found that the restoration of lysosomal degradation function and autophagic flux induced by SCU treatment, manifested as decreased P62, LAMP1, and ubiquitinated protein levels, was destroyed by *CTSD* silencing. The results of LysoTracker DND-99 and LysoSensor DND-189 double staining revealed that the fluorescence signals of LysoSensor DND-189 improved after SCU treatment and were inhibited when *CTSD* was silenced (Figures 6J, K). These results strongly suggest that the effect of SCU on promoting lysosomal function and autophagic flux is highly dependent on the upregulation of CTSD levels. The ROS production results (Figures 6L, M) showed that the ROS accumulation was inhibited by SCU treatment and was increased when *CTSD* was silenced at the same time. Moreover, under OGD/OGR conditions, LDH release rate significantly reduced after SCU treatment, which were reversed if *CTSD* was stably knocked down (Figure 6N). Overall, these findings demonstrate that SCU treatment protects endothelial cells against I/R injury by upregulating CTSD levels.

### 3.7 Scutellarin suppressed cardiac I/R injury through upregulation of CTSD

**FIGURE 7:**
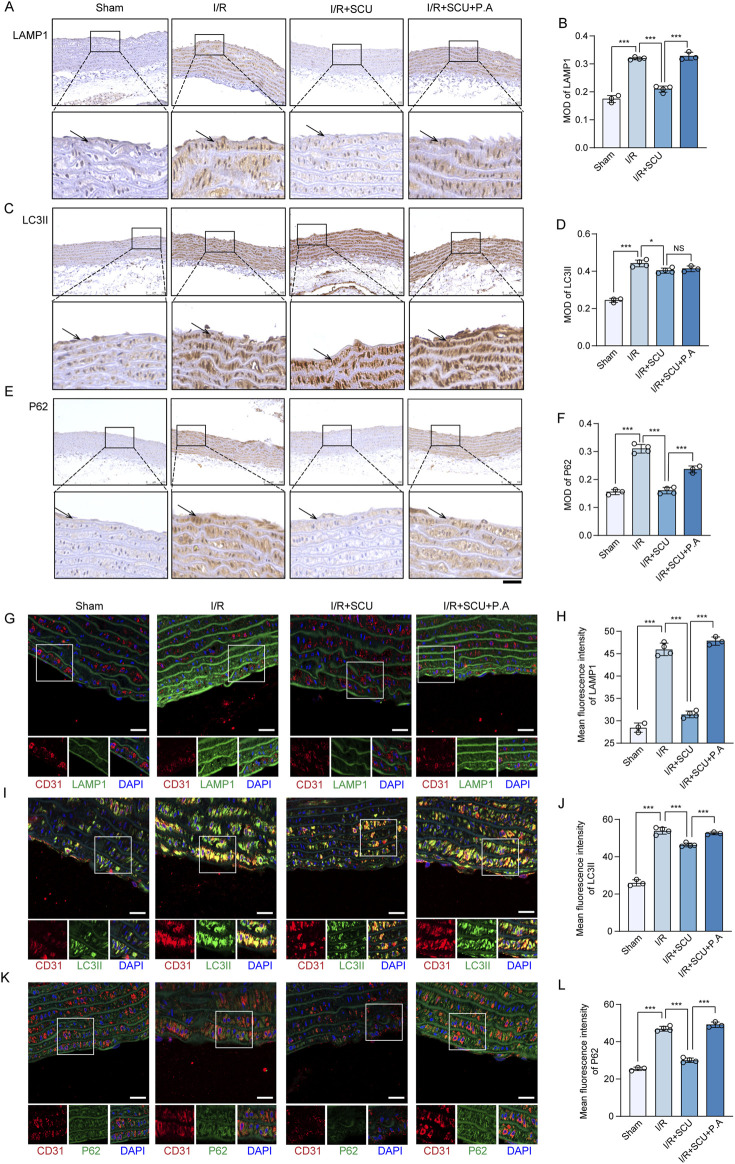
CTSD inhibition destroy the effects of Scutellarin in promoting autophagy-lysosomal function. **(A)** Representative IHC images and **(B)** quantification of LAMP1 in the aortic vascular tissues of sham, I/R, I/R+SCU and I/R+SCU+P.A rats (n = 4). **(C)** Representative IHC images and **(D)** quantification of LC3II in the aortic vascular tissues of sham, I/R, I/R+SCU and I/R+SCU+P.A rats (n = 4). **(E)** Representative IHC images and **(F)** quantification of P62 in the aortic vascular tissues of sham, I/R, I/R+SCU and I/R+SCU+P.A rats (n = 4). Scale bars, 100 μm. **(G)** Representative IF images showing staining of vascular endothelial cells with CD31 (Red) and LAMP1 (Green) in sham, I/R, I/R+SCU and I/R+SCU+P.A rats (n = 4). Scale bars, 20 μm. **(H)** Quantification of LAMP1 levels in CD31-stained positive cells (n = 4). **(I)** Representative IF images showing staining of vascular endothelial cells with CD31 (Red) and LC3II (Green) in ham, I/R, I/R+SCU and I/R+SCU+P.A rats (n = 4). Scale bars, 20 μm. **(J)** Quantification of LC3II levels in CD31-stained positive cells (n = 4). **(K)** Representative IF images showing staining of vascular endothelial cells with CD31 (Red) and P62 (Green) in ham, I/R, I/R+SCU and I/R+SCU+P.A rats (n = 4). Scale bars, 20 μm. **(L)** Quantification of P62 levels in CD31-stained positive cells (n = 4). I/R, ischemia/reperfusion; SCU, scutellarin; P. A, pepstatin A; LAMP1, lysosomal associated membrane protein 1; LC3II, MAP1A/1B light chain 3B; CD31, platelet endothelial cell adhesion molecule-1; MOD, mean of optical density.

**FIGURE 8:**
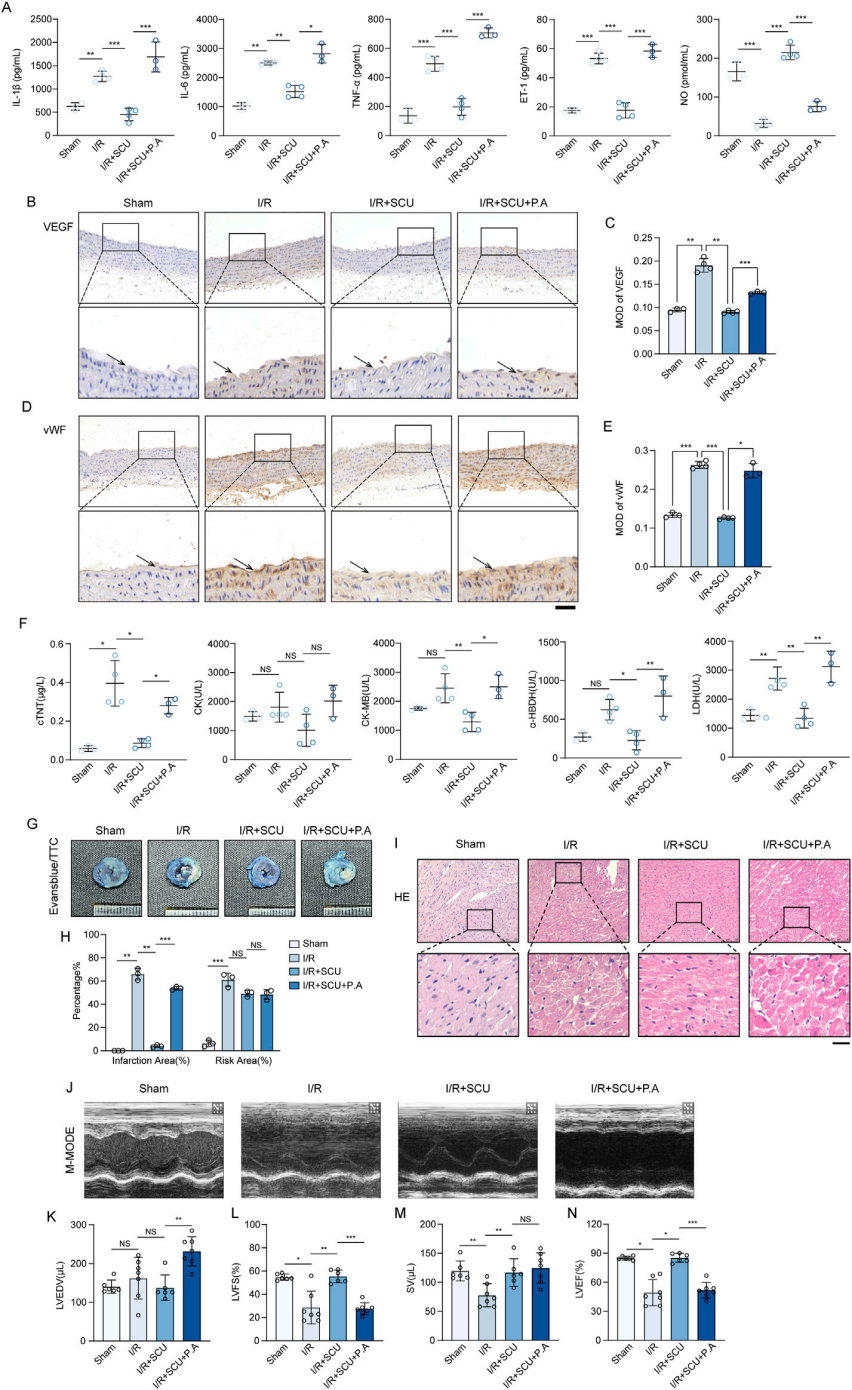
Scutellarin suppressed cardiac I/R injury through upregulation of CTSD. **(A)** Quantification of serum IL-1β, IL-6, TNF-ℵ, ET-1 and NO levels in sham, I/R, I/R+SCU and I/R+SCU+P.A groups (n = 4). **(B)** Representative IHC images and **(C)** quantification of VEGF in the aortic vascular tissues of sham, I/R, I/R+SCU and I/R+SCU+P.A rats (n = 4). **(D)** Representative IHC images and **(E)** quantification of vWF in the aortic vascular tissues of sham, I/R, I/R+SCU and I/R+SCU+P.A rats (n = 4). **(F)** Quantification of serum cTNT, CK, CK-MB, LDH and a-HBDH levels in sham, I/R, I/R+SCU and I/R+SCU+P.A groups (n = 4). **(G)** Representative photos of evans blue/TTC staining and **(H)** quantification of risk area and infarction area sizes (n = 3). **(I)** Representative micrographs of HE staining. Scale bars, 100 μm. **(J)** Representative acquisition of M-mode images. **(K–N)** Quantification of LVEDV (μL) **(K)**, LVFS (%) **(L)**, SV (μL) **(M)** and LVEF (%) **(N)** in sham, I/R, I/R+SCU and I/R+SCU+P.A groups (n = 7 for each group). Data shown are means ± SD; _NS_ *P*> 0.05, *P< 0.05, **P< 0.01 and ***P< 0.001 vs. the indicated group. I/R, ischemia/reperfusion; SCU, scutellarin; P. A, pepstatin A; IL-1β, interleukin 1 beta; IL-6, interleukin 6; TNF-α, tumor necrosis factor alpha; ET-1, Endothelin 1; NO, nitric oxide; VEGF, vascular endothelial growth factor; vWF, von willebrand factor; cTNT, cardiac troponin T; CK, creatine kinase; CK-MB, creatine kinase-MB; LDH, lactate dehydrogenase; a-HBDH, alpha hydroxybutyrate dehydrogenase; TTC, triphenyltetrazolium chloride; HE, Hematoxylin-eosin; LVEDV, left ventricular end systolic volume; LVFS, left ventricular fraction shortening; SV, stroke volume; LVEF, left ventricular ejection fraction.

**FIGURE 9:**
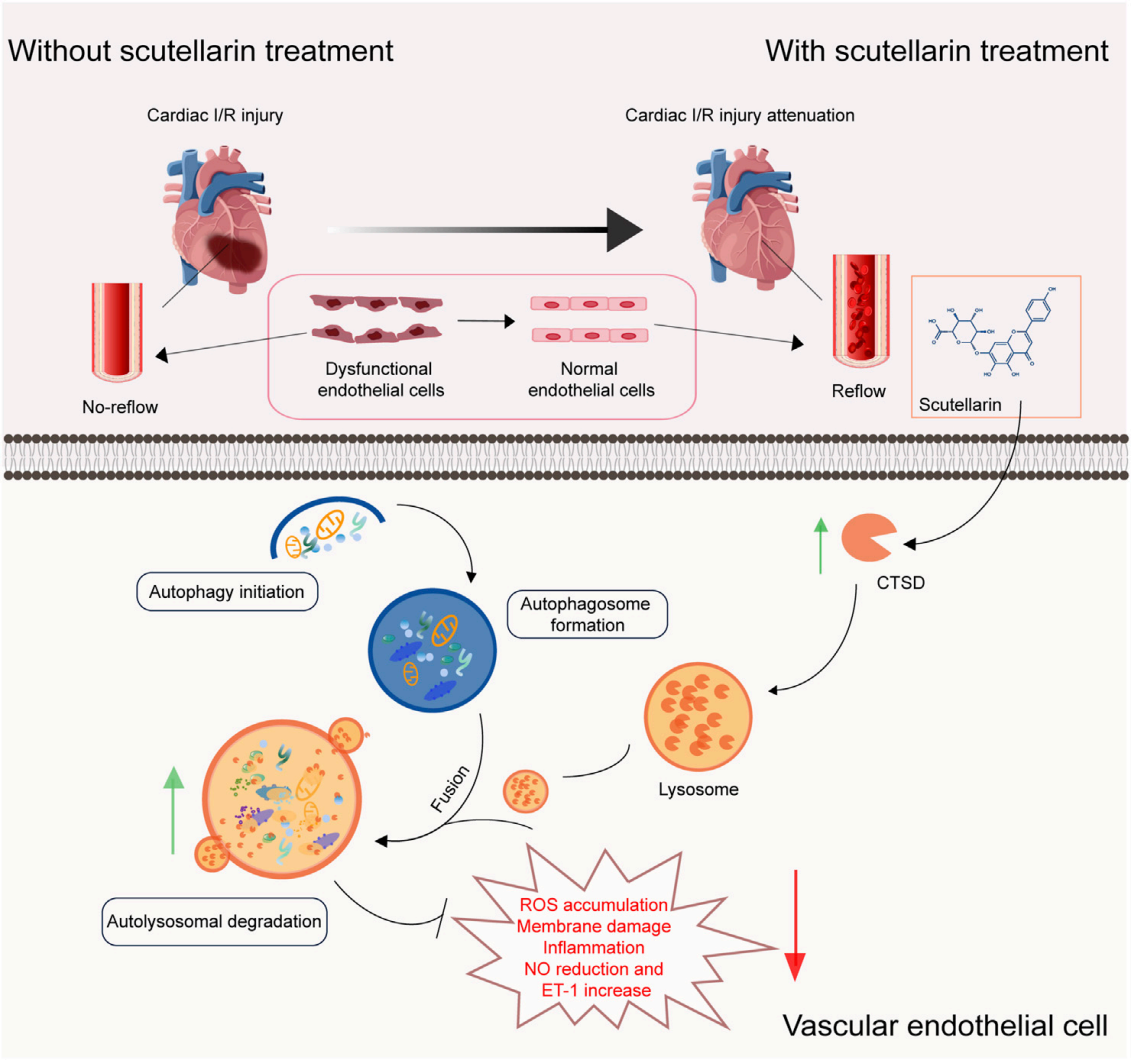
Scutellarin ameliorates ischemia/reperfusion-mediated endothelial dysfunction through upregulating CTSD expression to rescue autophagy-lysosomal function. **(A)** Schematic model illustrating that scutellarin alleviating I/R mediated-endothelial dysfunction, promoting vasodilation and blood reperfusion, inhibiting myocardial infarction and improving cardiac function through upregulating CTSD levels to rescue autophagy-lysosomal function in endothelial cells. CTSD, cathepsin D; ROS, reactive oxygen species; ET-1, Endothelin 1; NO, nitric oxide.

Inspired by the *in vitro* observations above, we then further tested whether the maintenance of CTSD-dependent autophagy-lysosomal function was required for the anti-I/R injury effects of SCU *in vivo*. We used P.A, which is a potent selective inhibitor of CTSD, to inhibit the activity of CTSD (McAdoo et al., 1973). Notably, IHC staining data (Figures 7A–F) showed that P.A pretreatment almost completely abrogated the promotion effect of lysosomal flow and autophagic flux induced by SCU pretreatment in I/R rats, as indicated by the re-elevation of P62 and LAMP1 levels. Additionally, IF staining data (Figures 7G–L) suggested similar changes in the aortic endothelium as IHC staining, indicating that CTSD is necessary for SCU to improve lysosomal function and autophagic flux. On this basis, it was observed that SCU led to a notable reduction in the serum levels of inflammatory factors (IL-1β, IL-6 and TNF-α) and ET-1, along with an increase in NO levels, while these effects were abolished by pretreatment with P.A at the same time (Figure 8A; Table 2). IHC results (Figures 8B–E; Table 2) showed that the beneficial effect of SCU on reducing VEGF and vWF levels was significantly offset by P. A. pretreatment. Furthermore, as shown in Figure 8F and Table 1, pretreatment with P.A abolished the reductions in the serum levels of myocardial enzymes (cTNT, CK, CK-MB, LDH and α-HBDH) induced by SCU pretreatment. In addition, Evans blue/TTC staining (Figures 8G, H) showed that SCU pretreatment slightly decreased the risk area and significantly reduced the infarct size in I/R rats, while these effects were reversed after P.A pretreatment. HE staining (Figure 8I) showed that SCU could inhibit the microscopic view of a recent myocardial infarction in I/R rats, which was abolished by pretreatment with P.A. Finally, echocardiography was performed to evaluate cardiac function. The echocardiography data (Figures 8J–N; Table 3) showed that I/R rats with SCU pretreatment had significantly improved contractile performance, manifested as increased LVFS%, SV, and LVEF%, compared to the I/R group. However, the improvement induced by SCU was almost completely destroyed by P.A pretreatment. Collectively, these findings indicate that the upregulation of CTSD and the maintenance of CTSD-dependent autophagy-lysosomal function are required for SCU to protect the endothelium and myocardium from I/R injury (Figure 9).

**TABLE 3:** Echocardiography results.

Indicator	Sham (n = 6)	I/R (n = 7)	I/R + TH (n = 7)	I/R + SCU L (n = 7)	I/R + SCU H (n = 6)	I/R + SCU + P.A (n = 7)
LVEDV (μL)	140.5 ± 7.0	162.1 ± 20.3^NS^	143.2 ± 10.3^NS^	147.8 ± 19.1^NS^	137.7 ± 13.4^NS^	198.6 ± 18.7^‡‡^
LVFS (%)	54.7 ± 1.1	28.6 ± 5.3^*^	44.7 ± 4.3^NS^	50.9 ± 3.8^†^	55.4 ± 2.3^†^	35.8 ± 4.6^‡‡‡^
SV (μL)	119.6 ± 7.0	77.6 ± 7.5^*^	104.7 ± 8.9^NS^	115.9 ± 9.5^†^	116.8 ± 9.7^†^	124.7 ± 9.9^NS^
LVEF (%)	85.0 ± 0.8	52.2 ± 7.1^*^	73.8 ± 4.8^NS^	80.5 ± 3.7^†^	85.3 ± 1.9^†^	64.9 ± 5.4^‡‡‡^

Note. Data shown are means ± SD. ^NS^ *P > *0.05, ^*^ *P* < 0.05, ^**^ *P* < 0.01 and ^***^ *P* < 0.001 vs. the Sham group; ^NS^ *P > *0.05, ^†^ *P* < 0.05, ^††^ *P* < 0.01 and ^†††^ *P* < 0.001 vs. the I/R group; ^NS^ *P > *0.05, ^‡^ *P* < 0.05, ^‡‡^ *P* < 0.01 and ^‡‡‡^ *P* < 0.001 vs. the I/R + SCU H group. I/R, ischemia/reperfusion; TH, tirofiban hydrochloride; SCU L, scutellarin low; SCU H, scutellarin high; P.A, pepstatin A; cTNT, cardiac troponin T; LVEDV, left ventricular end systolic volume; LVFS, left ventricular fraction shortening; SV, stroke volume; LVEF, left ventricular ejection fraction.

## 4 Discussion

Cardiac I/R injury is a major concern in the cardiovascular field. However, few medications are currently available to prevent I/R injury, and its treatment of I/R injury remains mainly symptomatic treatment. We believe that the key to resolving the problem of cardiac I/R injury is the inhibition of I/R-mediated endothelial dysfunction. Endothelial cells are mainly anaerobic metabolites that have a high tolerance to hypoxia, but are very sensitive to reperfusion after hypoxia (Zheng et al., 2022). I/R can cause structural damage and endothelial cell dysfunction via multiple mechanisms, further aggravating myocardial injury. During I/R, the massive production of ROS directly induces mPTP opening and Ca^2+^ overload, thereby increasing mitochondrial permeability, damaging mitochondrial structure and function, and subsequently activating the inflammatory response of endothelial cells, leading to endothelial dysfunction (Adameova et al., 2022; Wang et al., 2020; Zheng et al., 2022). Dysfunctional endothelial cells can induce inflammatory cells to adhere to or migrate to the injured vascular endothelial surface and release numerous inflammatory factors by upregulating the expression of adhesion molecules on their surfaces (Chang et al., 2021). Additionally, because of an increase in inflammatory substances, the production of ROS also increases, leading to a further decrease in the number of mitochondria with normal physiological functions. This causes endothelial dysfunction, eventually creating a vicious cycle. Moreover, high intracellular ROS levels can oxidize NO to peroxynitrous oxide, thereby reducing the bioavailability of NO and impairing vasodilation (Chang et al., 2021). And ET-1 can be secreted by injured endothelial cells to promote vascular contraction (O'Brien and Shivgulam, 2023). Finally, this series of pathological changes in endothelial cells leads to microcirculation impairment and the no-reflow phenomenon, ultimately aggravating myocardial I/R injury. Thus, pharmacotherapy of I/R-mediated endothelial cells is essential.

Accumulating evidence has demonstrated the beneficial role of Chinese herbal medicines in treating cardiovascular diseases. Recent meta-analyses demonstrate that both curcumin and tanshinone IIA exhibits excellent cardioprotective effects on myocardial I/R injury, as evidenced by reduced infarct size and improved cardiac function, and this cardioprotective effect may be related to their antioxidant, anti-inflammatory and anti-apoptotic properties (Li et al., 2023; Zhu et al., 2023; Zeng et al., 2023; Yan et al., 2023). A preclinical study by researchers from Jilin University show that ginsenoside Rc can ameliorated myocardial I/R injury by activating silent information regulator 1 (SIRT1) to reduce mitochondrial oxidative stress and apoptosis (Xue et al., 2023). Zhang et al. find that stachyose exerts a protective effect against myocardial I/R injury by targeting both ferroptosis and pyroptosis pathways (Zhang et al., 2024). These studies collectively highlight the potential of natural-derived compounds in mitigating cardiac I/R injury and the importance of understanding their underlying pharmacological mechanisms, particularly in regulating oxidative stress and cell death. SCU, a natural flavonoids compounds found in *E. breviscapus*, has garnered increasing attention for its therapeutic potential, including antioxidant, anti-inflammatory, anti-fibrotic, and lipid-lowering effects (similar to the Hua Zhuo effect of traditional Chinese medicine), and has been formulated into a variety of dosage forms for the treatment of cardiovascular and cerebrovascular diseases, such as atherosclerosis, coronary heart disease, and cerebral insufficiency (Nie et al., 2024). Clinical trial data by JIA et al. show that in AMI patients undergoing PCI, the combination of SCU injection effectively reduces oxidative stress and inflammation, and improved myocardial function. Furthermore, the incidence of major adverse cardiovascular events (MACE), including recurrent myocardial infarction, cardiac death, target vessel revascularization, and severe heart failure, is also reduced in SCU combined treatment group (Jia and Bai, 2019). A recent clinical study by researchers from Kunming Medical University find that the combination of SCU treatment and routine treatment after coronary artery bypass grafting (CABG) can improve the patency rate of saphenous vein bridge (SVG) and the left breast inside arteries (LIMA), preventing cardiac function, reducing ventricular remodeling without significantly increase the risk of bleeding (Peng, 2023). Additionally, SCU has been reported to reduce myocardial infarct size and restore cardiac function in I/R rats (Xu et al., 2020). In this study, we found that SCU pretreatment significantly inhibited myocardial injury in I/R rats, reduced myocardial infarct size, and improved cardiac function, indicating the ability of SCU to prevent cardiac I/R injury. Notably, through Evans blue/TTC staining, we found that not only was the infarction area reduced, but the risk area in I/R rats with SCU pretreatment was also slight decreased. We analyzed that SCU pretreatment may help maintain the integrity of the cardiac vascular in I/R rats, indicating that SCU also has a vascular protective effects. In this regard, we hypothesized that SCU could act on endothelial cells, significantly improving endothelial function, thereby promoting vasodilation, microcirculation recovery, and timely restoration of blood supply. This helps prevent extensive myocardial infarction and decline in cardiac function. Consistent with this hypothesis, we observed that SCU pretreatment significantly inhibited cellular structural damage, ROS accumulation, inflammation, NO reduction, ET-1 elevation and increase in the expression levels of VEGF and vWF *in vivo* and *in vitro* experiments.

Although the mechanisms by which I/R mediates endothelial dysfunction are complex, it is generally believed that high intracellular ROS levels are the primary factors leading to endothelial dysfunction. Autophagy is a highly conserved cellular metabolic process, and only a small amount of it is typically observed. Autophagy is activated and participates in the regulation of cellular homeostasis only when cells are stimulated by the internal and external environments. Excessive ROS production can act as signals to activate autophagy. Studies have demonstrated that enhanced autophagy can be detected in both *in vitro* and *in vivo* I/R injury models (Xu et al., 2023; Zeng et al., 2022; Cai et al., 2022). Studies have shown that enhanced autophagy can protect myocardial cells against I/R injury by the timely removal of damaged mitochondria and inhibition of cell apoptosis (Tong et al., 2020; Chen et al., 2021). In addition, autophagy cleans and degrades oxidized or dysfunctional proteins into amino acids and small molecules, which provide raw materials for the synthesis of new proteins, thus playing a cytoprotective role (Liu et al., 2023). However, the role of autophagy in cardiac I/R injury remains unclear. Some studies have found that excessive autophagy leads to the accumulation of autophagosomes in cells, thereby inducing autophagic apoptosis (Ali et al., 2022; Chen et al., 2023), and leading to cardiac remodeling induced by pressure overload and heart failure. Our prior work showed that autophagic degradation was inhibited while autophagic initiation was intact in the endothelial cells exposed to OGD/OGR (Zhuang et al., 2023). Further studies have found that impaired autophagic flux is harmful and could be a cause of I/R-mediated endothelial dysfunction. Therefore, instead of focusing on regulating occurrence of autophagy, ensuring the quality of autophagy and restoring autophagic flux may be more important in reducing cardiac I/R injury. Previous study has shown that SCU can prevent cisplatin-induced autophagy inhibition by enhancing LC3Ⅱ/I and autophagy-related protein 7 (ATG7) and inhibiting P62, thereby combating cisplatin-induced renal toxicity and protecting the kidneys (Sun et al., 2019). Moreover, a study found that after treating human lung cancer cells with SCU *in vitro*, cellular autophagy was enhanced and cell proliferation was inhibited (Cao et al., 2019). Studies have demonstrated that SCU can promote the phosphorylation of extracellular signal-regulated kinase 1/2 (ERK1/2) and inhibit protein kinase B (AKT) phosphorylation, thus inducing autophagy (Cao et al., 2019; Sun et al., 2018). In this study, we observed that although SCU pretreatment did not further promote autophagic initiation, it significantly increased autophagic flux, thus protecting endothelial cells against I/R injury.

The proper functioning of lysosomes is crucial for ensuring smooth autophay. Lysosomal dysfunction can induce the deposition of atypical protein aggregates, thereby causing cell death and disease progression (Gerenu et al., 2021; Wu et al., 2021). This has been repeatedly demonstrated in many studies of neurodegenerative diseases. In the present study, we found that SCU treatment protected endothelial cells from I/R injury by restoring autophagic flux. Considering the importance of lysosomes in maintaining autophagic flux, we hypothesized that SCU might have a protective effect on lysosomal function. LAMP1, a lysosomal membrane protein, plays a crucial role in the merging of autophagosomes with lysosomes (Levine and Kroemer, 2019). Our data showed that LAMP1 displayed an increasing trend both *in vitro* and *in vivo* in the endothelial dysfunction model, which was consistent with that of LC3Ⅱ/Ⅰ and P62 levels, suggesting a potential connection with the buildup of autolysosomes. Additionally, ubiquitinated proteins accumulated in endothelial cells, indicating dysfunctional lysosomal degradation in OGD/OGR-exposed endothelial cells. However, we observed that SCU treatment noticeably inhibited the accumulation of LAMP1 and ubiquitinated proteins, revealing that SCU can rescue lysosomal degradative dysfunction induced by I/R. As complementary proof, LysoTracker and LysoSensor staining confirmed that the acidic environment within the lysosomes was disrupted under I/R conditions, which led to the inability of the proteases to function properly, ultimately damaging the lysosomal degradative function. SCU treatment restored the acidic environment within the lysosomes, manifested as increased fluorescence signals of LysoSensor DND-189 after treatment with SCU. Considering these findings, we conclude that SCU can rescue the lysosomal degradative function in endothelial cells, thereby restoring impaired autophagic flux, which should be at least one of the pathways by which SCU inhibits I/R-mediated endothelial dysfunction. Thus, promoting the repair of lysosomal degradative function and the restoration of autophagic flux is expected to be a new mechanism of SCU in the prevention and treatment of cardiac I/R injury.

Maintenance of lysosomal degradative function depends on a series of hydrolases, including proteases, glycosidases, esterases, and nucleases, which are responsible for the catabolism of specific substrates. Lysosomal enzymes also play important roles in protein processing, the maintenance of lysosomal homeostasis, cell death, and immune responses. Owing to defects in lysosomal enzymes, the corresponding substrate cannot be degraded and accumulates in the lysosomes in large quantities, resulting in the disturbance of cell metabolism and related signaling pathways (Kuk et al., 2021). Among lysosomal enzymes, cathepsins, which are responsible for the degradation of proteins and peptides, have received the most attention. A growing number of studies have found that these genes are closely associated with aging and neurodegenerative diseases (Drobny et al., 2022). CTSD plays a crucial role in regulating lysosomal degradation. Wu et al. found that CTSD deficiency can induce lysosomal dysfunction in frontotemporal dementia (FTD) mice, and subsequently lead to the accumulation of myelin fragments, eventually promoting the occurrence and development of the disease (Wu et al., 2021). Decreased CTSD protein levels or the loss of protease activity can impair autophagy-lysosomal function and induce the intracellular accumulation of proteins associated with disease development, which are involved in the pathological processes of many different types of diseases, including neurodegenerative diseases (Bunk et al., 2021), stroke (Hossain et al., 2021), and fatty liver (Jeong et al., 2021). Consistent with these findings, our prior work demonstrated that CTSD protein levels and enzyme activity in endothelial cells simultaneously decreased after OGD/OGR treatment, and this reduction in CTSD protein levels was the main cause of lysosomal degradation dysfunction and impaired autophagic flux. In contrast, CTSD supplementation effectively improved lysosomal function and increased autophagic flux, thereby protecting endothelial cells from I/R injury (Zhuang et al., 2023). In this study, we were pleasantly surprised to observe that SCU treatment can significantly restored CTSD protein levels and increased its enzyme activity and found that the inhibition of CTSD almost completely abrogated the protective effects of SCU in I/R injury both *in vivo* and *in vitro*, suggesting that CTSD is essential for SCU in antagonizing I/R-mediated endothelial dysfunction and cardiac I/R injury.

However, there are several shortcomings in this study: Firstly, although we found that SCU pretreatment could reduce the serum ET-1 levels, increase the serum NO levels, and decrease the myocardial infarction area in I/R rats, indicating that SCU pretreatment may be beneficial for vasodilation and reperfusion, more direct evidence, such as changes in coronary artery diameter and coronary blood flow, is needed for further validation. However, due to technical limitations in our current experimental setup, we were unable to perform these measurements. Specifically, the instruments used in our study did not allow for the direct visualization or measurement of coronary vessels. In our follow-up studies, we will explore collaborations with institutions that have the advanced imaging technologies and expertise such as intravascular ultrasound (IVUS) or optical coherence tomography (OCT) and doppler flowmetry, to perform these measurements to further confirm our findings; Secondly, in this study, we performed IHC and IF staining on aortic vascular tissues to verify the changes in proteins such as LC3II, P62, LAMP1, and CTSD observed in the *in vitro* endothelial cell I/R injury model. Under the guidance of professional pathologists, we limited the observation range to ensure that our analysis primarily focused on the cells on the vascular lumen surface, mainly endothelial cells. However, due to the limitations of the dyeing method and the instruments used, we were unable to definitively prove that the observed changes were confined solely to endothelial cells. To address these limitations, in our future experiments, we plan to directly isolate endothelial cells from blood vessels and confirm the expression of the proteins of interest in endothelial cells using techniques such as flow cytometry and Western blot to further validate our findings and provide stronger evidence for our conclusions; Thirdly The present study was limited by the fact that the molecular pathway of SCU-mediated upregulation of CTSD in endothelial cells was not explored further. Our results showed that although the levels of CTSD increased after SCU treatment, there was no significant change in CTSD mRNA levels, suggesting that SCU may upregulate CTSD levels via post-transcriptional regulation, rather than transcriptional regulation. We also found that SCU significantly enhanced the stability of CTSD and inhibited its degradation, indicating a potential regulatory mechanism that SCU may regulate CTSD levels by inhibiting protein degradation. While our current study provides initial evidence of SCU’s post-transcriptional regulation of CTSD, a more detailed mechanistic analysis is necessary to fully understand this regulatory pathway. Future studies will focus on identifying the specific molecular mechanisms involved, including the role of protein post-translational modifications and protein-protein interactions in this process, to further clarify the molecular mechanism of SCU in antagonizing I/R injury and provide more evidence for the application of SCU in the prevention and treatment of I/R injury.

## 5 Conclusion

In summary, our data strongly demonstrate that SCU can resist I/R-mediated endothelial dysfunction and facilitate the recovery of microcirculation and blood reflow, thereby combating cardiac I/R injury. Its protective effects are achieved by the direct upregulation of CTSD levels and its ability to rescue autophagy-lysosomal function. These findings suggest that SCU may be a promising therapeutic agent for the prevention and treatment of cardiac I/R injury. Further large-scale preclinical trials and randomized controlled clinical trial are needed to confirm these findings.

## Data Availability

The original contributions presented in the study are included in the article/Supplementary Material, further inquiries can be directed to the corresponding authors.
